# Magnetoencephalography Dimensionality Reduction Informed by Dynamic Brain States

**DOI:** 10.1111/ejn.70128

**Published:** 2025-05-12

**Authors:** Annie E. Cathignol, Lionel Kusch, Marianna Angiolelli, Emahnuel Troisi Lopez, Arianna Polverino, Antonella Romano, Giuseppe Sorrentino, Viktor Jirsa, Giovanni Rabuffo, Pierpaolo Sorrentino

**Affiliations:** ^1^ Faculty of Biology and Medicine University of Lausanne Lausanne Switzerland; ^2^ School of Engineering and Management Vaud HES‐SO University of Applied Sciences and Arts Western Switzerland Yverdon‐les‐Bains Switzerland; ^3^ Institut de Neurosciences des Systèmes Aix‐Marseille Université Marseille France; ^4^ Department of Engineering Università Campus Bio‐Medico di Roma Rome Italy; ^5^ Institute of Applied Sciences and Intelligent Systems, National Research Council Pozzuoli Italy; ^6^ ICS Maugeri Hermitage Napoli Naples Italy; ^7^ Department of Medical Motor and Wellness Sciences University of Naples “Parthenope” Naples Italy; ^8^ DiSEGIM, Department of Economics, Law, Cybersecurity, and Sports Sciences University of Naples Parthenope Nola Italy; ^9^ Department of Biomedical Sciences University of Sassari Sassari Italy

**Keywords:** brain dynamics, dimensionality reduction, magnetoencephalography, neuronal avalanches, PHATE algorithm, resting state

## Abstract

Complex spontaneous brain dynamics mirror the large number of interactions taking place among regions, supporting higher functions. Such complexity is manifested in the interregional dependencies among signals derived from different brain areas, as observed utilising neuroimaging techniques, like magnetoencephalography. The dynamics of this data produce numerous subsets of active regions at any moment as they evolve. Notably, converging evidence shows that these states can be understood in terms of transient coordinated events that spread across the brain over multiple spatial and temporal scales. Those can be used as a proxy of the ‘effectiveness’ of the dynamics, as they become stereotyped or disorganised in neurological diseases. However, given the high‐dimensional nature of the data, representing them has been challenging thus far. Dimensionality reduction techniques are typically deployed to describe complex interdependencies and improve their interpretability. However, many dimensionality reduction techniques lose information about the sequence of configurations that took place. Here, we leverage a newly described algorithm, potential of heat‐diffusion for affinity‐based transition embedding (PHATE), specifically designed to preserve the dynamics of the system in the low‐dimensional embedding space. We analysed source‐reconstructed resting‐state magnetoencephalography from 18 healthy subjects to represent the dynamics of the configuration in low‐dimensional space. After reduction with PHATE, unsupervised clustering via K‐means is applied to identify distinct clusters. The topography of the states is described, and the dynamics are represented as a transition matrix. All the results have been checked against null models, providing a parsimonious account of the large‐scale, fast, aperiodic dynamics during resting‐state.

The study applies the PHATE algorithm to source‐reconstructed magnetoencephalography (MEG) data, reducing dimensionality while preserving large‐scale neural dynamics. Results reveal distinct configurations, or ‘states’, of brain activity, identified via unsupervised clustering. Their transitions are characterised by a transition matrix. This method offers a simplified yet rich view of complex brain interactions, opening new perspectives on large‐scale brain dynamics in health and disease.

## Graphical abstract

The study applies the PHATE algorithm to source‐reconstructed MEG data, reducing dimensionality while preserving large‐scale neural dynamics. Results reveal distinct configurations, or ‘states’, of brain activity, identified via unsupervised clustering. Their transitions are characterized by a transition matrix. This method offers a simplified yet rich view of complex brain interactions, opening new perspectives on large‐scale brain dynamics in health and disease.

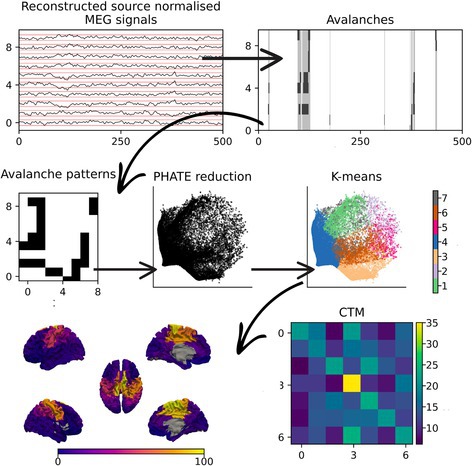

**Abbreviations**
AAL: automated anatomical labelling
ECG: electrocardiogram
EEG: electroencephalography
EOG: electrooculogram
fMRI: functional magnetic resonance imaging
ICA: independent component analysis
MEG: magnetoencephalography
MRI: magnetic resonance imaging
PCA: principal component analysis
PHATE: potential of heat‐diffusion for affinity‐based transition embedding
SD: standard deviation
TE: echo time
TI: inversion time
TR: repetition time
t‐SNE: t‐distributed stochastic neighbour embedding
UMAP: uniform manifold approximation and projection

## 1 Introduction

Coordination among brain regions is needed to generate appropriate behavioural responses for cognition and to be ready during resting state (Uddin 2021). The interactions occurring among brain regions are mirrored by statistical correlations between signals representing the activities of the regions. In the healthy brain, the dynamics of these dependencies evolve into complex patterns, continuously rearranging themselves, even during the resting state (i.e. when a subject is not engaged in a specific task). More specifically, recent evidence suggests that interactions among regions rapidly build and elapse as quickly, generating corresponding transient events of activities that spread over specific spatiotemporal trajectories. Over time, different subsets of brain areas participate in these transient coordinated events (Sorrentino et al. 2021a). In other words, the interactions at a large scale do not occur continuously but, rather, intermittently (Sorrentino et al. 2023). The statistics of these events are generally described by fat‐tailed distributions (e.g., power laws) (Palva et al. 2013; Hindriks and Tewarie 2023). In the context of critical dynamics, these transient collective fluctuations have been understood as ‘neural avalanches’ (Shine et al. 2019). Regardless of whether these transient events genuinely capture the presence of a dynamics operating near a critical point, converging evidence shows that the dynamics of these large‐scale states is relevant for cognitive activities and is altered in case of neurological ailments. From there, we can ask ourselves, do patterns of activities spread preferentially along specific spatial trajectories? Furthermore, is there a specific temporal order among such trajectories? Along these lines, several studies focused on the identification of altered brain dynamics via the definitions of microstates (Michel and Koenig 2018). Unlike these studies, we focused here on the dynamics of the aperiodic transient events of activities. We hypothesised that the subset of brain regions recruited by each event are not random but, rather, transient topographies are explored in a structured order with nontrivial transition probabilities. We leveraged source‐reconstructed magnetoencephalography data, which offer high spatiotemporal resolution, from 18 healthy young adults, and described the sequence of events by their activation ‘patterns’, corresponding to the set of brain regions participating in each event. Previous research has highlighted that patterns of neuronal avalanches effectively pinpoint the locations and moments where activities resonate (Sorrentino et al. 2023), which is related to the recruitment of brain areas during tasks (Corsi et al. 2024). Hence, feeding avalanche patterns to a state‐of‐the‐art dimensionality reduction technique might help classify brain states and capture the main features of the high‐dimensional avalanche patterns sequence (Cunningham and Yu 2014; Williamson et al. 2019). While a plethora of dimensionality reduction techniques exists, they often seem to fail preserving both the local and global similarity of the large‐scale brain activities (e.g., with t‐distributed stochastic neighbour embedding (t‐SNE) (Arora et al. 2018)). potential of heat‐diffusion for affinity‐based transition embedding (PHATE), on the other hand, preserves these aspects of the data structure in the low‐dimensional representation, providing a smoother account of the evolution of the system and, hence, of the sequence of the patterns (Moon et al. 2019). Therefore, we utilised this algorithm to reduce the dimensionality of the data. The low‐dimensional representations were then grouped into a number of ‘states’ using the k‐means algorithm, and the topography of each state was described. Finally, we demonstrated that neither the states we defined nor the transitions between them are expected by chance alone. When the network dynamics are destroyed, for example, by temporal randomisation, the observed patterns also disappear.

## 2 Material and Methods

### 2.1 Data

#### 2.1.1 Participants

A total of 18 right‐handed native Italian speakers participated in the study. Participants were required to meet specific criteria: They should be free from significant medical conditions, substance abuse and medications that could affect magnetoencephalography/electroencephalography (MEG/EEG) signals. Additionally, they needed to be free from major systemic, psychiatric or neurological illnesses and show no evidence of brain damage on routine magnetic resonance imaging (MRI) scans. The study was approved by the Ethics Committee ASL‐NA1 centro (Prot.n.93C.E./Reg. n.14‐17OSS), and all participants provided written informed consent.

#### 2.1.2 MRI Acquisition

Brain images were acquired using a 1.5 Tesla MRI scanner (SIGNA, GE Healthcare) with a 3D T1‐weighted Magnetisation‐Prepared Gradient‐Echo BRAVO sequence. Imaging parameters included a TR of 8.2 ms, TE of 3.1 ms and TI of 450 ms, with a voxel size of 1 × 1 × 1 mm^3^ and a 50% partition overlap across 324 sagittal slices covering the entire brain.

#### 2.1.3 MEG Acquisition

Magnetoencephalographic data were collected using a 163‐magnetometer MEG system in a magnetically shielded room (AtB Biomag UG, Ulm, Germany). Preprocessing followed established protocols. Four coils and four reference points (nasion, right and left preauricular points and apex) were digitised using the Fastrak (Polhemus) prior to acquisition. Brain activity was recorded for ~7 min with eyes closed, divided in two halves to reduce drowsiness. The head position was measured at the start of each segment. Data were sampled at 1024 Hz and filtered with a fourth‐order Butterworth band‐pass filter between 0.5 and 48 Hz. Electrocardiogram (ECG) and electrooculogram (EOG) were recorded during acquisition. MATLAB 2019a and the Fieldtrip toolbox 2014 were used for preprocessing.

### 2.2 Data Processing

#### 2.2.1 Preprocessing

To reduce environmental noise, principal component analysis (PCA) was employed. Noisy channels were manually removed through visual inspection by an experienced rater. Supervised independent component analysis (ICA) was utilised to remove ECG and EOG artefacts from the MEG signals. Trials free from artefacts or excessive noise were selected for further analysis. Typically, around 1 min of data was discarded due to artefacts.

#### 2.2.2 Source Reconstruction

The MEG data were coregistered with native MRI scans (Sorrentino et al. 2018). A modified spherical conductor model served as the forward model. Voxels in the MRI were labelled according to the automated anatomical labelling (AAL) atlas, focusing on 78 cortical regions and 12 subcortical regions (Table ST1). A linearly constrained minimum variance beamformer computed 90 time series, each corresponding to one region of interest, at a sampling frequency of 1024 Hz. An experienced rater visually inspected the reconstructed sources. The source‐reconstructed signals were then downsampled to 256 Hz.

#### 2.2.3 Avalanche Pattern

For each subject, each source‐reconstructed MEG time series was standardised (z‐scored) and binarised based on a threshold fixed at three SD, following (Corsi et al. 2024). Furthermore, we analysed the reliability of clustering across varying z‐thresholds. Specifically, we examined the entropy of the clusters based on different thresholds and the correlation between the clusters obtained using different thresholds (Figure SP1). Our analyses confirmed the robustness of the clustering approach across different z‐thresholds. The entropy measures indicated a rather stable threshold selection, while high correlations between results at different thresholds demonstrated consistency in cluster assignment. Together, these findings confirm that our results are not dependent on a specific choice for the threshold.

Deviations above the threshold, marked by ones in the binarised signals, tend to co‐occur across subsets of brain regions, which suggests the presence of spatiotemporal correlations and collective behaviours operating across different cortical areas (Müller and Meisel 2023).

Based on the binarised signals, we defined neuronal avalanches as starting when at least one region is above threshold, and ending when no region is above threshold (Corsi et al. 2024). Then, for each avalanche, we established an *avalanche pattern* as a vector of size *N*, with *N* representing the number of brain regions, containing 1 if a region was recruited during the avalanche (at any point and for any duration), and 0 otherwise. In other words, we analysed the spatial structure of each avalanche, while disregarding the internal structure.

**FIGURE 1:**
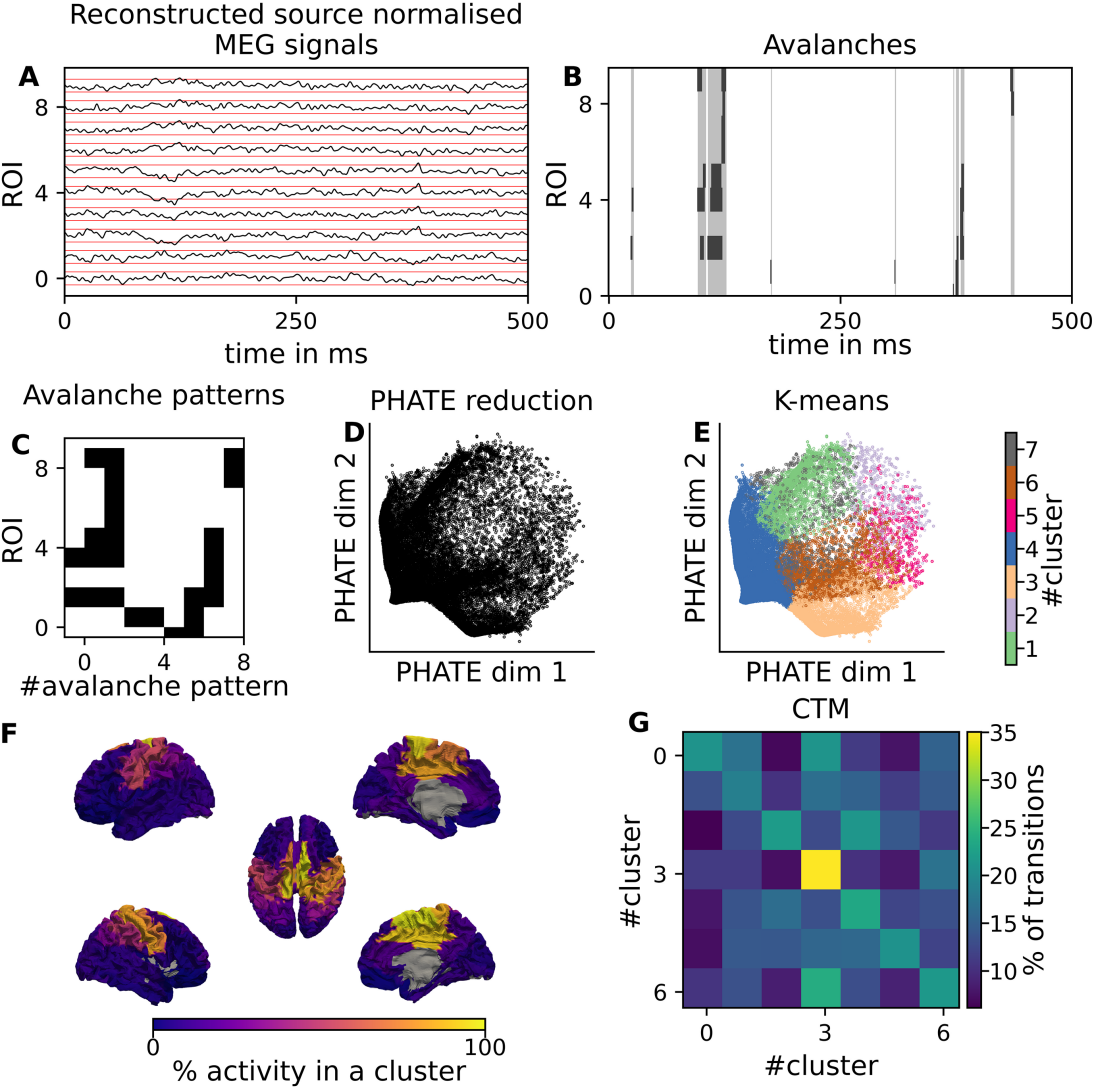
Overall Pipeline steps. Magnetoencephalography (MEG) source‐reconstructed signals are used to detect neural activity (A). After normalising each signal and z‐scoring them, transient coordinated events of brain activity across multiple channels (i.e., neuronal avalanches) are identified (B). Each avalanche is then vectorised to define an ‘avalanche pattern’, capturing the active regions during the event and encapsulating the spatial and temporal information about brain activity (C). The dimensionality of the data is reduced using a technique called PHATE (D) (potential of heat‐diffusion for affinity‐based trajectory embedding), which helps visualise and interpret high‐dimensional data in a low‐dimensional space. Finally, K‐means clustering is applied to the primary PHATE components to extract the final clusters (E), resulting in the identification of seven clusters characterised by their average activity vector (e.g., the Cluster 6) (F). The probability matrix of changing clusters (G).

**FIGURE 2:**
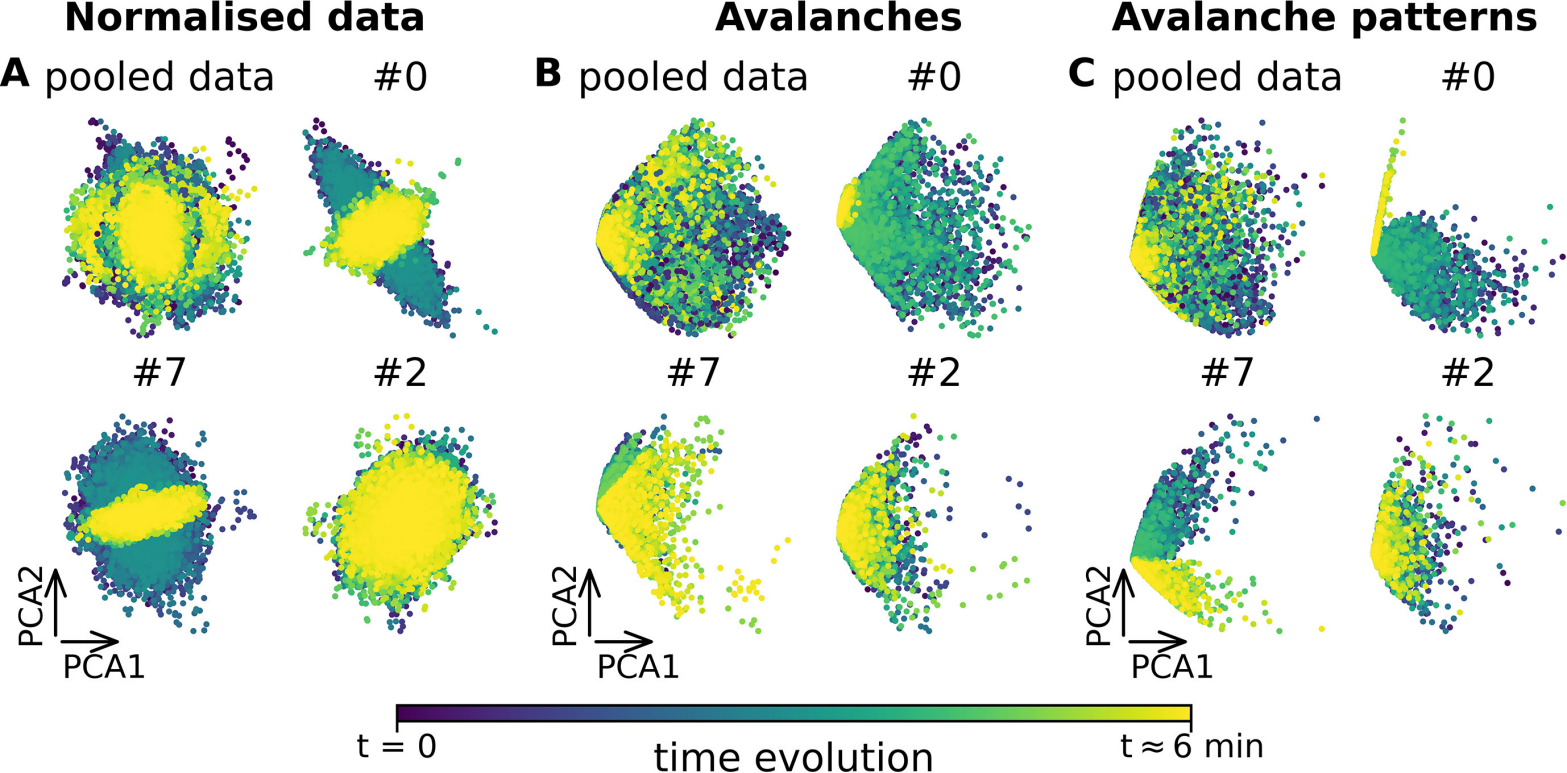
Low‐dimensional reduction using PCA. The picture shows the time evolution of the system by subject in PCA space for the different steps of the pipeline and the low‐dimensional reduction using PCA detection starting from normalised data (A), avalanche activity (B) and avalanche patterns (C). In each subplot, each dot represents the evolution of the whole system brain state at each instant along the first (PCA1) and second (PCA2) dimension. (The colour gradient from dark purple to yellow indicates the time evolution from the beginning to the end of the recording) for pooled data and the subject 0, 1 and 2 as examples. We notice how focusing on avalanches most details are discarded from the data, but this reveals an underlying structure that it is already possible to capture using PCA. It is interesting to consider that the duration (t end, around 6 min) varies for each subplot, as different subjects exhibit different durations.

We then concatenated all the unique avalanche patterns and used these as the input for the dimensionality reduction algorithm, PHATE. Figures 1 and 2 provide, respectively, an overview of the pipeline and an overview of the various stages of the input data in a low‐dimensional reduction with PCA.

#### 2.2.4 PHATE

PHATE is an algorithm designed to visualise multidimensional data by a nonlinear dimensionality reduction that preserves, in the low dimensions, the local and global structure of the data. This algorithm is described in detail in Moon et al. (2019). In a nutshell, the first step is the application of a PCA where only the most informative components are retained. In this study, the first five components were identified as the most significant based on the explained variance (percentage of the total variance) and their prominence in the cumulative variance plot (see Section 2.3 for more details, as well as Figure SP2). The procedure to preserve local and global structures in low dimensions relies on calculating the distances between each avalanche pattern (a point in high‐dimensional space) using cosine similarity (Figure SP3). These distances are then transformed into a Markov‐normalised affinity matrix using a kernel function. For our specific application, the kernel was configured to consider the five nearest neighbours, with an ɑ‐decay of 1 (see below Section 2.3.2). Based on this, a diffusion probability is calculated and used directly to embed the cosine distance with multidimensional scaling into a three‐dimensional space.

#### 2.2.5 Unsupervised Machine Learning: K‐Means Clustering

**FIGURE 3:**
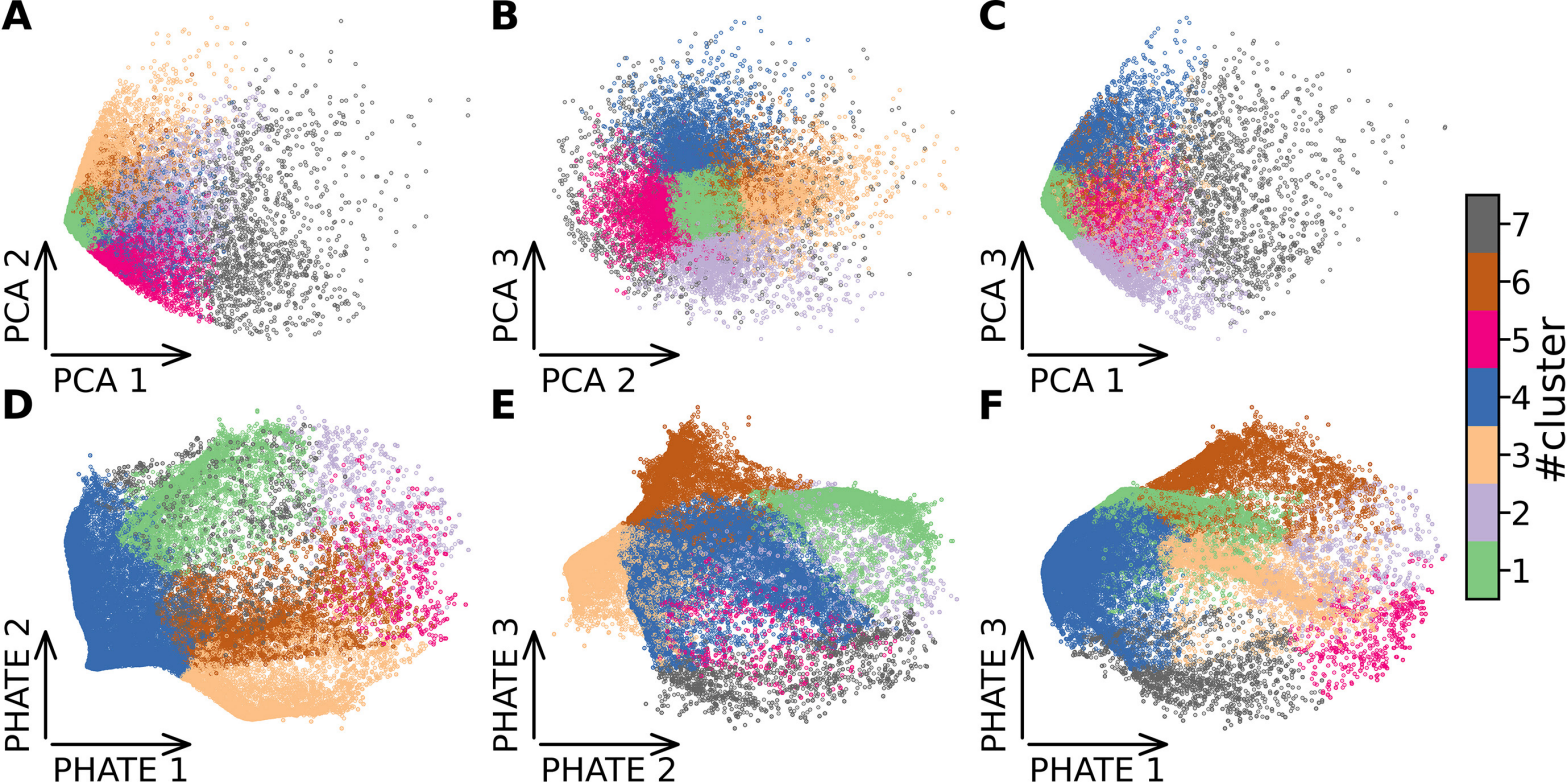
Comparison of low dimensions of PCA and PHATE. The process of dimensionality reduction is done using the potential of heat‐diffusion for affinity‐based trajectory embedding (PHATE) algorithm. Initially, PCA is used to reduce the data into five principal components, the three firsts are shown in the scatter plots (PC1, PC2 and PC3 in panels A, B and C, respectively). The data points are then further processed through PHATE, which reveals a more nuanced structure, effectively separating the clusters based on intrinsic data properties. This transformation is visualised in a two‐dimensional scatter plot of the first three axes (PHATE 1, PHATE 2 and PHATE 3 in panels D, E and F, respectively). It highlights distinct clusters, which are subsequently categorised using K‐means clustering for seven clusters (indicated by various colours). For all panels, each point represents an avalanche pattern sorted on the axes of the primary components (PCA or PHATE).

After the dimensionality reduction via PHATE (or via a simple PCA as for Figure 3), we classified the points in low dimensions using unsupervised K‐means clustering. This algorithm operates iteratively to group data into clusters. The first iteration randomly initiates a number (here *k* = 7) of cluster centroids. Each point in the training dataset is then assigned to the nearest centroid based on Euclidean distance (the method for selecting the number of centroids, *k*, is detailed in the Section 2.3 that follows). In the subsequent steps, each centroid is moved to the mean of the points assigned to it. After one iteration is finished, the next iteration will again assign each training sample to the closest cluster centroid and move those centroids again according to the mean of the points. Either a maximum number of iterations is fixed or the algorithm will continue until the change of position of the centroids is under a predetermined ‘tolerated’ value.

#### 2.2.6 Transition Between Clusters

**FIGURE 4:**
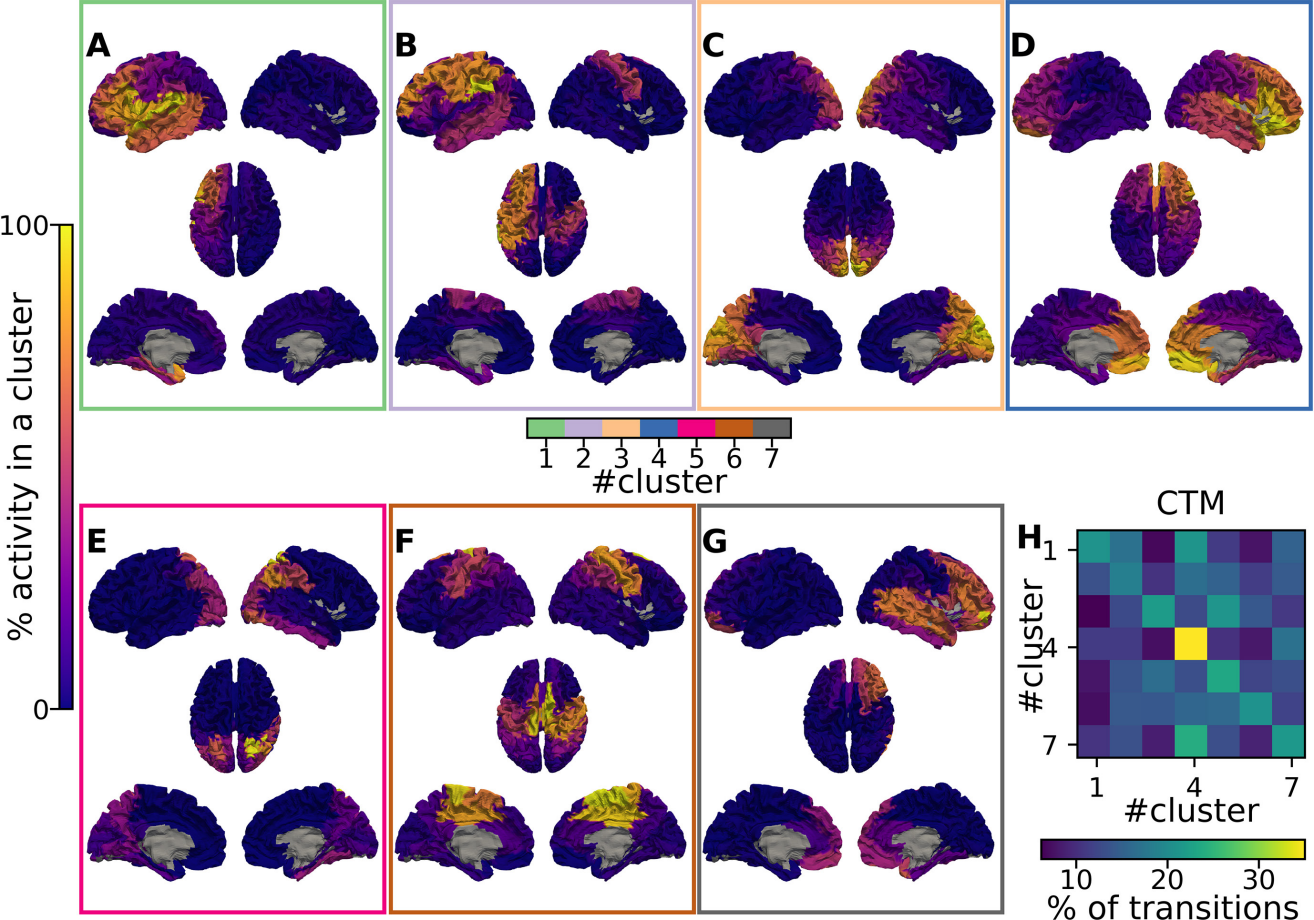
Output of the pipeline on brain images and matrices. The pipeline is applied on the avalanche patterns to reduce the dimension with PHATE and then cluster each avalanche with K‐means algorithms. The result of the pipeline is the average activity of each cluster of our output projected on brain images (A, B, C, D, E, F and G). More precisely, it shows the projection on brain images of the statistical activation (yellow) or inactivation (dark blue) of cortical regions for each cluster. The second output of the cluster is (J) the probability of transition between each cluster, that is, the transition matrix.

Based on the clusters identified by the k‐means algorithm, we labelled each avalanche pattern and computed the transition probability matrix for each subject, capturing the transitions between clusters (Figure 4H). Additionally, we computed a global transition matrix that aggregates the avalanche patterns across all subjects. Notice that the latter includes some ‘false transitions’ due to the concatenation of avalanche patterns belonging to different subjects. However, due to the low number of ‘false transitions’, this should not bias the estimated probability.

### 2.3 Sensibility Analysis

Sensibility analysis was applied to evaluate the uncertainty about the most important parameters used in our pipeline: the number of components in the PCA and the ɑ‐decay for the PHATE algorithm, as well as the number of clusters for the k‐means.

#### 2.3.1 Assessment of the Number of Components in the PCA of PHATE Algorithm

To determine the number *C* of components in the first step of PHATE, we plotted the PCA explained variance (percentage of the total variance) relative to the number of components. For this, the evolution of the cumulative explained variance was plotted against the number of components of the PCA (see Figure SP2). The optimal number of components was selected such that no visual significant difference in explained variance was observed by using *C* or *C + 1* components, ensuring that adding more than 5 components does not significantly improve the explained variance.

#### 2.3.2 Assessment of the ɑ‐Decay Parameter for the PHATE Algorithm

In order to fix the PHATE parameter ɑ‐decay of 1, we first used empirical tests, trying to reduce it in order to capture the smallest local structures. However, a minimal value of the decay can be estimated by calculating the maximal distances between neighbours provided by the K‐nearest neighbour algorithm (Figure SP3). This estimation is based on the calculation of the kernel, which requires to provide a significant weight with the desired number of neighbours.

#### 2.3.3 Assessment of the Number of Clusters in the K‐Mean Clustering (For Different Kernels of PHATE)

We assessed the number of clusters by looking at three different graphical methods: the elbow measure, the silhouette method and the gap statistic (K‐means Cluster Analysis).

The elbow method is based on a graph showing the within‐cluster‐sum‐of‐square (WCSS) values for different numbers of centroids (*k*). We described the gain in explained variance as a function of the number of clusters (Figure SP4). The point of flexion provides an estimate for the optimal number of clusters.

The silhouette method (Figure SP4) is based on plotting the average silhouettes of our observations for various numbers of centroids. A good clustering is indicated by a high average silhouette. The optimal k is the one that maximises the average silhouette.

The gap statistic method (Figure SP4) is based on comparing the difference, that is, *gap*, between the expected total intracluster variation and the observed one for a specific *k*. This is then repeated for different values of *k* and plotted as a function of the number of clusters. The higher the gap, the more a particular number of clusters provides a parsimonious account of the variance in the data.

To visualise the topography of the clusters, we computed how much each region is recruited in the patterns belonging to each cluster (summing the number of times each region is active in patterns belonging to a given cluster). This way, we describe cluster‐specific topographies.

### 2.4 Validation

**FIGURE 5:**
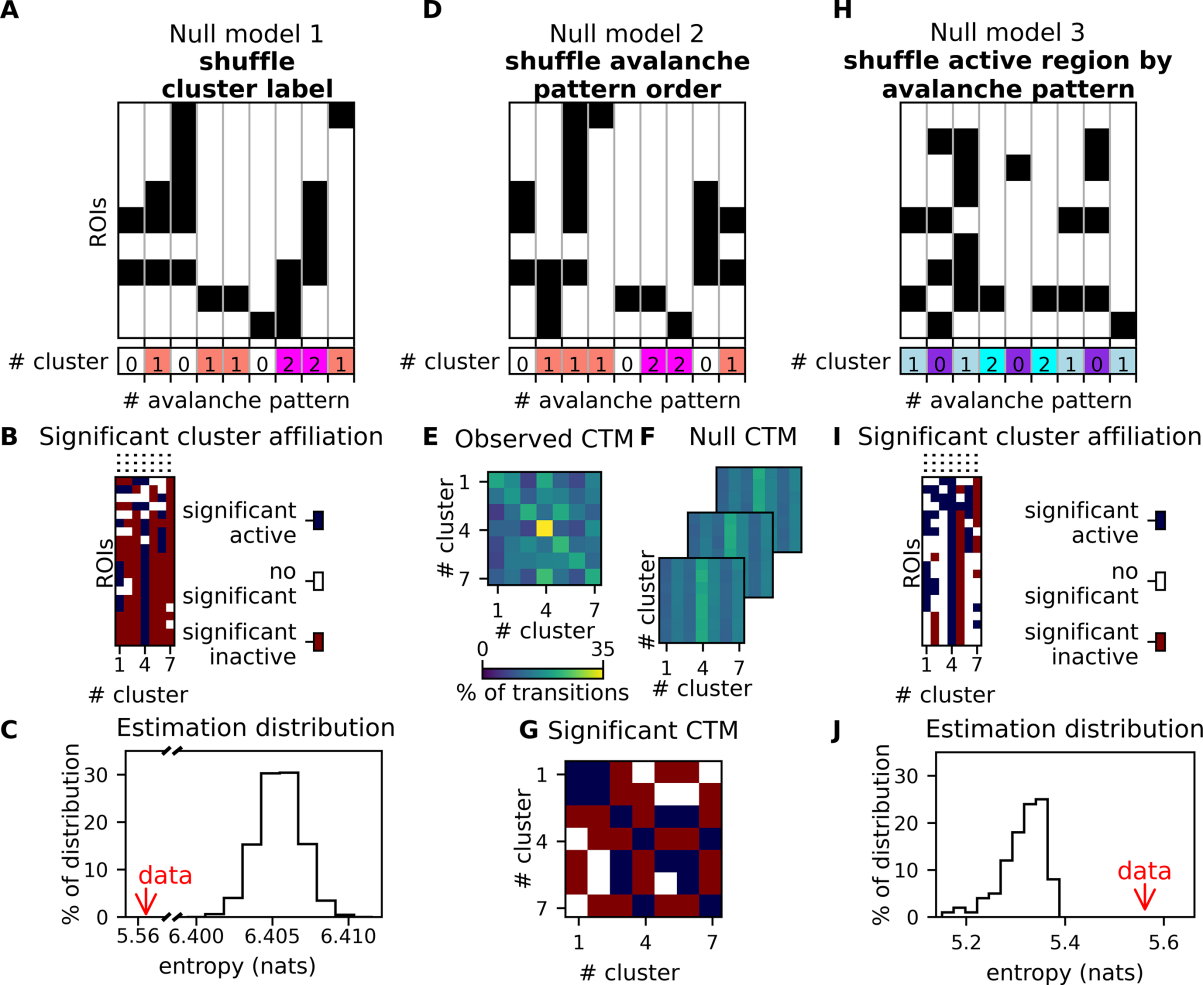
Robustness analyses. The figure highlights the three methods to check the robustness of our findings, based on null models. The first solution (null model 1) mixes cluster labels (A) and observes the entropy distribution (C) resulting from such shuffling (in black) versus our original one (red arrow). The panel B (as the following panel I) displays the partial vectors of all clusters (the full vectors are displayed in Figure SP6). In other words, they represent the sum of avalanches belonging to the cluster, resulting from the association of each avalanche to a specific cluster. More specifically, those panels show the significance of the probabilities of an avalanche recruited above chance, that is, ‘activated’ (blue), excluded above chance, that is, ‘inactivated’ (red), or not significantly recruited nor excluded (white) from a cluster. The second null model (null model 2) is based on shuffling the avalanche patterns' order (D) to test the robustness of the transition. Matrices E, F and G display, respectively, the probabilities of transitions between each cluster for our original data, the significance of the probabilities of a cluster and the shuffled ones. The last one (null model 3) is to shuffle each active region in each avalanche pattern (H) and check the robustness of the dimensional reduction, computing the entropy distribution of the cluster label shuffled output (black distribution) and entropy measure of our original output (red arrow) (J).

The robustness of the results was evaluated by comparing our observations to null models. In particular, we analysed the robustness of (1) the activation pattern of each cluster (Figure 5A,B,C), (2) the transition prediction between clusters (Figures 5D,E,F, G, SP5) and (3) the dimensionality reduction (Figure 5H,I,J).

The null model hypothesis suggests that any observed differences in the studied characteristic from a dataset are attributable to chance. The null model hypothesis suggests that any observed differences in the studied characteristic from a dataset are attributable to chance. Hence, for each robustness analysis (point 1, 2 and 3 in parts 2.4.1, 2.4.2 or 2.4.3, respectively), we shuffled 10,000 times the data to obtain a null distribution of the features; each analysis requires a different way of randomising the data, resulting in a different ‘null dataset’. Other than the shuffling of the data according to the studied features, we performed for each null model, the same preprocessing, obtaining the avalanche patterns. Then, we applied the same PHATE and K‐means clustering algorithms. We kept the identical dimension and cluster numbers across the various methods that we are comparing, avoiding hyperparameter optimisation. This way, it is possible to directly compare the entropies that are yielded by the different methods.

Finally, we computed the entropy for the null models 1 and 3, and the percentage of significant transitions for the model 2. We then compared the features between the existing dataset and the random ones, demonstrating if a statistical property in the data was to be expected by chance.

#### 2.4.1 Robustness of the Activation Pattern of Each Cluster

First, based on the null model hypothesis, we wanted to test if the clusters obtained by our algorithm were informative and not random. As a result of the algorithm (PHATE and k‐means), each avalanche pattern was associated with a cluster. From this association, as explained above, we described the activity pattern of each cluster, that is, a vector containing the average activation of each brain region (e.g., brain plots in Figure 4). To check that the clusters were nonrandom, we computed the entropy of the cluster matrix by concatenating the $N_{c}$ cluster patterns, which resulted in an array x of length $M=90\cdot N_{c}$, where 90 is the number of regions. We compared the empirical entropy to the ones observed in the null cluster patterns. The entropy (in nats) of the array *x*, H(*x*), was calculated based on Shannon's equation (Shannon 1948):

$$H\left(x\right)=-\sum_{j=1}^{M}\left(x_{j}\times \ln\left(x_{j}\right)\right)$$

where $x_{j}$ corresponds to $\xi_{i,n}$, which is the percentage of activity of i‐th region inside the n‐th cluster and M is the length of the concatenated cluster vectors.

This way, we obtained the distribution of the entropies of the null data, against which we compared the entropy of the clusters we observed from nonrandom labelling (Figure 5C).

Comparing our original output with the null output we also verified, for each brain region in each cluster, if any given region was more (less) recruited in the patterns belonging to a particular cluster (Figures 5B and SP6). We did this by comparing the probability of any region being recruited in the observed clusters to the probability of the same regions being recruited in a pattern obtained after the 10,000 shuffling. We defined the significance threshold as 0.05.

#### 2.4.2 Robustness of Transition Prediction Between Clusters

For each subject, we tested whether the transitions between the clusters were statistically significant. This involved generating shuffled sequences of each subject's data to serve as a baseline for comparison. Transition predictions between clusters for each subject were then evaluated against these shuffled sequences (Figure SP7). The transition probabilities of the original sequence were compared to those derived from the corresponding shuffled sequences, computing the correlation between them and yielding a *p*‐value for each transition probability (Figure SP8). Looking at the resulting *p*‐value matrix for each subject, we could identify statistically significant transitions (both occurring more or less often than what is expected by chance alone) at a significance level of 0.05. The transition sequences per subject also enabled us to evaluate intersubjects variability (e.g., mean, standard deviation and coefficient of variation between the transitions).

Finally, we used the same method described in the paragraph above to determine whether the transitions between clusters were nonrandom across subjects, as predicted by the null model. This analysis involved comparing the transition probabilities from the concatenated sequences against those from 10,000 shuffled sequences, mirroring the approach used for individual subject data (Figure 5F). We calculated the *p*‐values for each transition probability in the concatenated sequences to assess statistical significance (Figure 5G). Similar to the individual analyses, significant transitions were identified based on a threshold of 0.05.

This way, we could estimate to what extent the pattern of the transitions is shared among subjects. Indeed, if the prediction of transition is robust between clusters, an additional analysis can be done to identify consistent cluster transitions across individuals. This consistency would suggest that rather than being the result of individual variances or random variation, the observed transitions are most likely indicative of the population under study.

#### 2.4.3 Robustness of Dimensionality Reduction

To quantify the effectiveness of the dimensionality reduction, we used again the null model hypothesis, this time shuffling active and nonactive regions within each pattern. To this end, we shuffled the labels of all regions—both active and inactive—within the input data for each avalanche prior to feeding it into PHATE for dimensionality reduction, and k‐means for clustering. Subsequently, we assessed the robustness of the dimensionality reduction by comparing the entropy of the low‐dimensional output obtained from the original data against the entropy distribution observed when starting from the 10,000 shuffled data. As described above, we verified, for each region and for each cluster, the probability of the activation or deactivation of the region if the null model hypothesis was true, providing an estimate of the confidence that the activations of any regions that are included in a given pattern are nonrandom (Figures 5H–J and SP6). We considered a statistical significance threshold of 0.2. The reduction of precision is due to the fact that the instances of the different clusters are not homogeneous (i.e., some clusters are present more often than others). As a consequence, the statistical power and the detectable effect size change across patterns.

#### 2.4.4 Benchmarking Against Other Dimensionality Reduction Techniques

In order to interpret the goodness of the performance of the PHATE algorithm, we carried out the same preprocessing and then utilised different algorithms to classify the data. In particular, we have used PCA and spectral clustering. For PCA, it corresponds to the first step of the PHATE algorithm. We chose this to compare our results against a level of preprocessing that is as close as possible to what is being fed to the nonlinear steps of the algorithm. On the other hand, we chose spectral clustering since both techniques rely on spectral methods to capture the intrinsic geometry of high‐dimensional data.

#### 2.4.5 Scalability Analysis: Application of the Algorithm to a Larger Sample Size

To further validate our findings, we replicated them utilising a larger cohort of 44 subjects. We examined the clusters retrieved in both cohorts and visualised the corresponding differences (Figure SP9). We also computed the average of the maximum correlation among the topographies of the clusters obtained in the two cohorts.

## 3 Results

### 3.1 Combining Neuronal Avalanches and Dimensionality Reduction to Characterise Brain Network Dynamics

Starting from the source‐reconstructed MEG signals (Figure 1A), we identified the sequence of neuronal avalanches through a binarisation process; (Figure 1B; see Section 2) and defined for each avalanche the set of regions that were recruited as an *avalanche pattern* (Figure 1C). Then, we fed the sequence of avalanche patterns to the PHATE algorithm to reduce their dimensionality (Figure 1D). This way, we preserved the spatial information about the patterns as well as their evolution over time. Applying the K‐means algorithm to the first components of PHATE (Figure 1E) allowed us to cluster the avalanche patterns into self‐similar groups. Averaging the avalanche patterns within each cluster resulted in characteristic brain networks with nontrivial topography (e.g., Figure 1F). Studying the original sequence of avalanche patterns, and labelling the patterns according to the corresponding cluster, we extracted the transition probability matrix, or Cluster Transition Matrix (CTM). We have also repeated the analysis using PCA as a dimensionality reduction technique, to compare the performance with that of PHATE. Note that neuronal avalanches are based on a binarisation algorithm that greatly reduce the amount of information in the data: Signals can only have 0 (‘inactive’) and 1 (‘active’) values, and the threshold for binarisation is set in such a way that only a small percentage of time points (e.g., 5%) is considered as active. Nonetheless, focusing on neuronal avalanches prior to applying dimensionality reduction techniques (i.e., PHATE and PCA) allowed us to extract valuable information regarding the topography and dynamics of brain states, while reducing the noise in brain data.

### 3.2 Focusing on Neuronal Avalanches Reveals Hidden Low‐Dimensional Structure

To analyse the advantages of reducing data informed by neuronal avalanche algorithm, we analysed the performance of dimensionality reduction techniques when applied to the source‐reconstructed data (‘source data’) (Figure 1A), to the avalanche activity (i.e., without averaging the activity within each avalanche; Figure 1B) or to the avalanche patterns (Figure 1C). We start by focusing on the PCA dimensionality reduction, projecting the brain activity (source‐reconstructed signals, avalanche activity or avalanche patterns) onto the first two PCA components (Figure 2). Feeding the avalanche patterns to the PCA (Figure 2C) highlights a more rich low‐dimensional data structure as compared to the normalised source data (Figure 2A) or to the avalanche activity (Figure 2B). In general, using the avalanche activity and the avalanches patterns (Figure 2B,C, respectively) improves the separation of the data points as compared to the normalised data (Figure 2A). Furthermore, as observed in Figure SP10, the use of avalanches highlights a common structure, removing the specificity of subjects and highlighting common structures. In the Supporting Information, we report the data reduction starting from the reconstructed z‐scored MEG source signals (Figure SP11), from avalanches (Figure SP12) and from the avalanches patterns (Figure SP13) for all the subjects, and for each of the 18 subjects individually. This suggests that the avalanche analysis can enhance the interpretability, as well as the visualisation of the underlying neuronal dynamics as compared to using normalised source data. In other words, focusing on the avalanche patterns provides a theoretically informed, yet data‐driven way to select only a handful of points in time and space, which are most significant. Similar trends were observed employing PHATE to reduce the dimensionality of the data (Figure 3; see also Figure SP14 where PHATE is applied to different levels of preprocessing).

### 3.3 PHATE Dimensionality Reduction Outperforms PCA on Brain State Identification

As explained in the methods, PHATE aims at preserving both the details and the high‐level structures that are present in the high‐dimensional representation of the data (Moon et al. 2019). The PHATE algorithm entails a preliminary dimensionality reduction step based on PCA, a heat‐diffusion process to help denoising the data, while preserving the manifold distances in the lower dimensional embedding space.

Here, we applied PHATE or PCA to subject‐specific avalanches patterns and extracted the main avalanche patterns clusters using K‐means (as in Figure 1D,E).

Compared to PCA, PHATE retrieved clusters that are better separated, displaying a clearer three‐dimensional structure (Figure 3). In fact, PCA fails to show a clearly identifiable number of clusters. The PHATE algorithm detected seven clusters, identifying more patterns in the data and, as a result, helping the clusterisation.

Figure SP4 displays the elbow, silhouette and gap statistics graphs for both the PHATE and the PCA reductions.

In addition, the cumulative activations of each region indicate that no specific region seems to drive the classification (Figure SP15). This would suggest that the clustering captures genuinely multivariate patterns.

### 3.4 Identification of Brain State Topographies Using PHATE Dimensionality Reduction

We visualised how often each brain region was recruited in each cluster, providing a topographical account of the clusters (Figure 1F—see Section 2 for more information). Figure 4, shows that the cluster‐specific topography varies sharply, with each cluster showing an interpretable pattern of regions that are preferentially recruited. On the one hand, in Clusters 3 (Figure 4C) and 5 (Figure 4E), the occipital regions are the most active. Clusters 2 (Figure 4B) and 4 (Figure 4D), on the other hand, display symmetrical activities in frontal regions, with minor involvement of the contralateral frontoparietal regions. The frontotemporal regions are symmetrically involved in Clusters 1 (Figure 4A) and 7 (Figure 4G), while Cluster 6 (Figure 4F) hinges around the sensorimotor regions. As seen, despite the clustering being data‐driven, the clusters are reminiscent of known anatomical/functional patterns, supporting the validity of the cluster‐specific topographies.

### 3.5 Large‐Scale Dynamics: Identification of Brain State Topographies

Studying the transition matrix between clusters (CTM; Figure 1G), we show that, despite neuronal avalanches being separated by long interavalanche intervals, their sequence is far from being a random process. Much like an earthquake has a higher probability of being followed by aftershocks, we observe that the transition to the same cluster (i.e., as if the cluster is recruited multiple consecutive times) is the most probable transition, as shown by the high values of the main diagonal in Figure 4H (or Figure 1G). This can be seen as evidence that the system possesses a memory about the previously recruited pattern. The clusters could thus be considered quasi‐stable in time. However, some intercluster transitions display a high probability of occurring. Overall, most transitions are occurring with a probability higher than chance‐level, as shown in Figure SP16. Similarly, Figure SP17 displays the mean, standard deviation and coefficient of variation of the transitions between clusters for all subjects. We observed that the mean of the diagonal remains quite stable, around 0.2, with little variability except for Cluster 3, which has higher mean and higher variability, and Cluster 1, which shows high variability. Other features could be observed as well: Cluster 6 has a tendency to transit to Cluster 3, while the latter has a tendency to be recruited multiple times or to transit to Cluster 6. Finally, Cluster 1 has a tendency to transit to Clusters 2 and 3, while Cluster 2 has a tendency to transit to Cluster 1.

However, when displayed across subjects (Figure SP8), the significance of the transition probability between clusters highlights a high variability. This observation, already visible in the matrices of transition probabilities across subjects (Figure SP7), could be explained by the short span of the recordings. However, Figure SP18 shows that the same transitions (i.e., between the same clusters) occur above/below chance across subjects. In other words, the dynamics we observed is consistent across subjects, despite the dimensionality reduction being performed independently for each.

### 3.6 Sensibility Analysis and Statistical Validation

The sensitivity analysis, which has been carried out to test the robustness of our results (see Section 2), involved the evaluation of the number of components for the PCA within the PHATE algorithm and the optimal number of clusters for the K‐means clustering. To this end, we considered various parameters and statistical or visual measures, such as the explained variance and the gap statistics.

#### 3.6.1 Assessment of the Number of Components in the PCA of PHATE Algorithm

The assessment of the number of components in the PCA, which is carried out as the first step of the PHATE algorithm (Figure SP2) showed a marked drop until the fifth component (included). In fact, the explained variance went from about 0.125 (percent of the total variance) with one component, to roughly 0.35 with five components. When including more than five components, there is only a modest increase of the explained variance. Therefore, we chose to proceed taking five components into account.

#### 3.6.2 Assessment of the Number of Clusters in the K‐Mean Clustering (For Different Kernels of PHATE)

We used three methods to help us define the number of clusters to classify the output of the PHATE algorithm: the elbow, the silhouette and the gap statistics (see Section 2). For each, we ran the PHATE algorithm, with a different number of clusters, followed by the K‐means algorithm. Then, we plotted the elbow, silhouette and gap statistic (Figure SP4). We aimed at observing whether, as the number of kernels and PCA components in PHATE increased, the number of clusters would also increase. The elbow and the silhouette graphical outputs were difficult to interpret and did not yield a clear cut‐off for choosing the number of centroids. However, the gap statistic indicated the optimal trade‐off at *k* = 7 clusters, which was selected for further analyses.

#### 3.6.3 Statistical Validation

To confirm the result of our analyses, the robustness of the results was assessed through various tests, as shown in Figure 5. These included (1) examining the informativeness of the clusters as compared to random associations (Figure 5A), (2) testing the significance of transitions between clusters against random dynamics (Figure 5D) and (3) evaluating the stability of the clusters across different subjects. Similarly, as already described, the robustness of the dimensionality reduction was verified by comparing the probability of a region being recruited (or not) in a given cluster, to what is observed given random clusters (Figure 5H).

In Figure 5C, it is shown that the entropy of the subject data is significantly lower than the entropy of random clustering. This indicates that the clustering generated by our unsupervised method is more structured than the random clustering. As shown in Figure 5B,C, the null model demonstrates that the initial clusters are unlikely to be due to chance and, instead, reflect the recurrent coordinated activations (de)activations of specific groups of brain regions.

Then, Figure 5E shows that the transition probability matrix (containing the probability of sequentially evolving from a state to another state) has higher values in the diagonal, as compared to the off‐diagonal transitions. However, these probabilities in the diagonal remain lower than what would be expected by chance alone (Figures SP5 and SP18). Furthermore, there are also preferential transitions between different states (i.e., some sequence of transitions occur more/less often than what is expected by chance). Lastly, according to the entropy plot in Figure 5J, we observe that the entropy of our cluster is significantly higher than the entropy distribution of the data in which the regions' status were shuffled. In other words, in this null model, where active and inactive regions were shuffled within patterns, no meaningful structure is present. Hence, the patterns of regional activation/deactivation within the clusters are unlikely to be random. Additionally, it shows that the dimensionality reduction preserves the structure of the manifold throughout the dimensionality reduction.

#### 3.6.4 Benchmarking Against Other Dimensionality Reduction Techniques

As mentioned, we compared the performance of the PHATE algorithm to PCA and Spectral clustering. To compare PHATE with spectral clustering, we applied both algorithms to the avalanche patterns using the same preprocessing and processing steps described in the main text. This included noise reduction, source reconstruction, avalanche pattern creation, application of the chosen algorithm (either PHATE followed by K‐means or Spectral clustering alone), entropy measurement and visualisation against brain images.

For a direct comparison, we first analysed the data using the same number of clusters (7) for both methods, a choice based on PHATE's peak performance. This approach enabled a clearer evaluation of similarities and differences between the two techniques. The resulting cluster outputs are presented in Figures SP19 and SP20.

To quantitatively compare clustering methods (PHATE, Spectral clustering and PCA) and different preprocessing steps (clustering applied to source‐reconstructed signals, normalised signals or avalanche patterns), we measured the entropy of the vectorised cluster matrices (e.g., the first two matrices in Figure SP19; see updated methods in the main text). Higher entropy values indicate more complex but potentially noisier clusters, whereas lower entropy values suggest greater structure but possible redundancy. Ideally, an optimal clustering approach balances structure and information richness, meaning intermediate entropy values reflect a well‐balanced state between order and disorder.

The performance of the three clustering algorithms, assessed through entropy measurements, is summarised in Figure SP21 and Table ST2. Our analysis indicates that both PHATE and spectral clustering perform comparably, producing more structured clusters (lower entropy) than PCA. Entropy values for these comparisons are reported in Table ST2. Additionally, we examined how entropy varies as a function of the number of clusters, with results presented in Figure SP20 and Table ST3.

Finally, we assessed the similarity of clusters obtained with PHATE, PCA and spectral clustering through correlation analyses. Specifically, we correlated the topographies of the seven clusters obtained with each method. As shown in Figure SP22, different algorithms retrieve similar structures to some extent (e.g., PHATE Cluster 2 is highly correlated with spectral clustering Cluster 6 and PCA Cluster 1) but also generate distinct, nonmatching clusters (e.g., PHATE Cluster 4 does not have a clear counterpart in spectral clustering or PCA). Overall, PHATE clusters show higher correlation with spectral clustering than with PCA. To quantify this, we computed the maximum correlation for each PHATE cluster with clusters derived from PCA and spectral clustering and then averaged these maximum correlations separately for the two methods. The results, reported in Table ST4, highlight the degree of similarity between PHATE and the other clustering approaches.

#### 3.6.5 Scalability Analysis: Application of the Algorithm to a Larger Sample Size

The comparison of retrieved clusters (Figure SP9) and their differences revealed consistent topographical patterns. The average of the maximal correlation among cluster topographies in the two cohorts is equal to 0.78.

## 4 Discussion and Conclusion

A converging body of evidence shows that brain activities evolve according to complex, nonlinear dynamics (Zalesky et al. 2014). Measures such as the fractional occupancy, dwell‐times and transition probabilities have been used to describe the dynamical properties of the data (Song et al. 2023; Munn et al. 2023; van der Meer et al. 2020). These recurrent activation modes are modulated by a number of physiological phenomena (Liparoti et al. 2024) and have been linked to health/disease (Cipriano et al. 2024; Lopez et al. 2024; Polverino et al. 2022; Sorrentino et al. 2021b), task performance (Corsi et al. 2024) or even consciousness (Breyton et al. 2024).

In this work, we utilise eyes‐closed source‐reconstructed MEG scans from 18 healthy young adults during resting‐state. Our work builds on recent evidence suggesting that nonlinear transient events, that is, neuronal avalanches, represent moments where regions interact creating transient configurations, ‘states’. From this starting point, we could reduce the data complexity through signal binarisation, which allowed us to identify transient coordinated events. Then, we applied the PHATE algorithm (Moon et al. 2019) to reduce the dynamics to lower dimensionality, and K‐means clustering to reduce the number of states by grouping the avalanche patterns.

Neuronal avalanches can be understood as quasi‐stable configurations—or attractors—of the dynamics (Shriki et al. 2013). As the brain's activity evolves, it alternates between periods of uncoordinated activity, where regions operate independently, and moments of transient coordination, corresponding to neuronal avalanches. Over time, the brain visits multiple attractors (avalanche patterns), resulting in metastable dynamics. Importantly, not all states are visited: At rest, certain regions are more consistently recruited in avalanches (‘coordinated’) than others. While brain dynamics are fine‐tuned and, as mentioned earlier, its disruptions are related to disease, the specific trajectories (i.e., the sequence in which these states are visited) in the healthy brain remain largely unknown. We hypothesised that the transitions between states follow a regulated (i.e., nonrandom) sequence of avalanche patterns, with the successive topographies of these recruited patterns exhibiting a distinct structure. To test this hypothesis, we compared our proposed methodology against various surrogate models to demonstrate that the observed patterns are unlikely to have arisen by chance.

Despite neuronal avalanches occurring in less than 5% of the time points (e.g., there are only 3 s spent in avalanche activity out of 1‐min recording), these events carry unique information about the brain dynamics. In fact, previous research focusing on the neuronal avalanche statistics, topography and propagation pathways showed the potential of these transient events as a marker of healthy brain dynamics (Breyton et al. 2024; Rucco et al. 2020; Arviv et al. 2019). Thus, focusing on the sequence of avalanche patterns before feeding the data into dimensionality reduction or clustering algorithms might be a neurophysiological‐principled way to reduce the computational demand and the noise while focusing on the ‘most relevant’ part of the data. Indeed, in this work, we showed reliable state identification by operating on the neuronal avalanche patterns, hence discarding the rest of the data. In order to achieve this, we employed dimensionality reduction techniques on the sequence of avalanche patterns.

The primary goal of dimensionality reduction is to shift through redundant information in complex, multimodal datasets without relying on predefined assumptions. This approach maps the effective information contained in the original features to a low‐dimensional feature space. In this context, the evolution of the activity over time is often described as the system evolving over a low‐dimensional surface (i.e., a manifold), rather than exploring the full high‐dimensional phase space (Idesis et al. 2023; Rossi‐Pool and Romo 2019).

When applied to the description of the evolution of neural dynamics, dimensionality reduction techniques like PCA, ICA, t‐SNE and uniform manifold approximation and projection (UMAP) are not explicitly designed to retain both local and global nonlinear relationships in the low‐dimensional representation. For instance, t‐SNE excels in visualising high‐dimensional datasets by minimising the Kullback–Leibler divergence between joint probabilities in the embedding and the original data, but its outcomes can vary due to the usage of gradient descent with random initialisation for optimising a nonconvex problem (Arora et al. 2018).

Hence, these techniques might not be best suited for data with multiple, nested spatiotemporal scales, such as MEG data. In contrast, as mentioned earlier, PHATE preserves both local and global structures across scales, constructing a diffusion‐based informational geometry that effectively captures manifold‐intrinsic distances, offering a detailed and scalable low‐dimensional embedding, which might be best suited for MEG data. Additionally, PHATE shows good denoising properties for clearer visualisation and provides robust, scalable visualisations that capture a broader range of patterns (Moon et al. 2019).

Furthermore, other techniques, such as time‐varying dynamic state models (e.g., DyNeMo (Gohil et al. 2022)), as well as Hidden Markov Modelling (HMM), might be compared to PHATE in future studies.

Regardless of the above, applying PHATE and K‐means clustering to MEG avalanche patterns from healthy individuals led to the identification of distinct clusters corresponding to specific brain networks. In particular, based on the cluster identified by the K‐means algorithm, we computed the transition probability matrix for each subject, capturing the transitions between clusters and also a global transition matrix that aggregates the avalanche patterns across all subjects. After testing the significance of transitions between the clusters, involving shuffled sequences of each subject's data to serve as a baseline for comparison, we found that the observed transitions are most likely indicative of the population under study and are not random and the transition to the same cluster is the most probable transition meaning that the system possesses a form of memory about the previously recruited pattern. Of note, it appears that certain states are preferred at the beginning of the recorded period, and some at the end. We speculate that this might be linked to infraslow dynamics that cannot be properly captured by our data.

On one hand, the comparison with PCA reduction (Figure 3A,B,C), demonstrates the effectiveness of PHATE in capturing the flow of neural activities, enhancing both the cluster identification and the visualisation of brain states. This approach was further validated through sensibility analysis and robustness testing, confirming the reliability of the findings (Sections 2.3 and 3.6.3). On the other hand, the comparison with spectral clustering, a technique that also utilises a spectral approach to capture the high‐dimensional geometry of the data, shows comparable performances. This talks to some stability of the retrieved patterns, which can be found, once avalanche patterns are extracted, with different algorithms. However, spectral clustering might be less suited for large datasets, as it requires the computation of all affinity matrices, which is demanding.

As per the generalisability of our findings, the replication of the main results on a different cohort is reassuring.

Dimensionality reduction techniques applied to resting‐state data suffer, in general, from the lack of validation about the ‘goodness’ of the clusters. To this end, we devised a large array of null models to extensively test both the spatial structure of the clusters, which is unlikely to emerge given a random clustering of the regions, and their evolution, which is not justified by a random sequence of the spatial clusters.

To our knowledge, this is the first study applying the PHATE algorithm to MEG data. In particular, the algorithm had been already applied to EEG (Zhang et al. 2023) and fMRI data (Busch et al. 2023; Iyer et al. 2022; Rieck et al. 2020; Busch et al. 2024), where it is shown that PHATE could visualise brain states and help identify altered brain dynamics in early stages of schizophrenia. While these studies have provided convincing evidence of the efficacy of PHATE (e.g., (Busch et al. 2024) with the T‐PHATE algorithm or (Iyer et al. 2022) combining data from several sources, including fMRI), none of the previous works focused on neuronal avalanches (in MEG data).

It would nonetheless be interesting to further investigate limitations of the PHATE algorithm regarding autocorrelating signals such as fMRI (Busch et al. 2023). Indeed, in such data, we may want to explore an approach combining PHATE with a model of signal autocorrelation. In addition, a study presents another limitation of PHATE algorithm (Wängberg et al. 2022), as discrete structures could be more accurately defined, although it also underlines PHATE's capability in preserving graph distances and local and global ones, assuming multiple tests of parameters are realised.

Moreover, we wish to stress that our model, while robust, is hard to validate, given the lack of available models that can reproduce realistic (i.e., large) functional repertoires of the aperiodic bursts. This problem is hard to circumvent. One future approach could be to seek external validation utilising data with different stimuli (as opposed to the current dataset, which is acquired during the resting state). If different stimuli would map to different areas of the low‐dimensional space, this would amount to an indirect validation of the appropriateness of our approach.

In the end, our study highlights the potential for such dimensionality reduction for MEG data as well as the potential outputs that can be obtained from this. In that regard, further studies on MEG data may benefit from using the PHATE algorithm.

Our findings also reveal well‐defined activation patterns, temporality and spatially distinct, expressed in terms of transient coordinated events, in the brain activity among healthy subjects. The clusters appear to be biologically meaningful, in that they either correspond to well‐defined anatomical structures (e.g., the left and right frontal lobes in Figure 4), or they are reminiscent of known functional clusters, such as the default mode network. Crucially, if one performs dimensionality reduction directly from the source data (i.e., not from the avalanche patterns), the algorithm fails to retrieve meaningful, nonrandom patterns. This can be observed in the Figure SP14, panel A, where one can visually appreciate the loss of performance in the data separation. To sum up, the dynamics of spontaneous activities, based on avalanche patterns, is constrained to prefer specific sequences and to go across other sequences less often than expected (given the relative dwell‐time across state, which is kept constant in the surrogates). Such constraints are preserved in the low‐dimensional representations. In conclusion, our work demonstrates that brain dynamics can be understood as a fine‐tuned sequence of intermittent nonlinear transient coordinated events (neuronal avalanches) that modify their spatiotemporal structure over time. The implications are manifolds, as nonlinear transient events are typically overlooked in favour of a more oscillatory perspective. This approach allows a theory‐driven, mathematically principled way to vastly reduce the complexity of the data. After this step, the deployment of advanced dimensionality reduction techniques successfully led to a parsimonious data‐driven description of the large‐scale dynamics.

## Author Contributions

**Annie E. Cathignol, Lionel Kusch, Giovanni Rabuffo and Pierpaolo Sorrentino**: conceptualisation and methodology. **Annie E. Cathignol, Lionel Kusch, Marianna Angiolelli, Giovanni Rabuffo and Pierpaolo Sorrentino:** simulations and data analysis. **Annie E. Cathignol, Lionel Kusch, Marianna Angiolelli, Giovanni Rabuffo and Pierpaolo Sorrentino:** formal analysis. **Annie E. Cathignol, Viktor Jirsa and Pierpaolo Sorrentino:** resources. **Annie E. Cathignol, Lionel Kusch, Marianna Angiolelli, Giovanni Rabuffo and Pierpaolo Sorrentino:** writing–original draft. **Annie E. Cathignol, Lionel Kusch, Marianna Angiolelli, Emahnuel Troisi Lopez, Arianna Polverino, Antonella Romano, Viktor Jirsa, Giovanni Rabuffo and Pierpaolo Sorrentino:** writing–review–editing. **Annie E. Cathignol, Lionel Kusch, Marianna Angiolelli, Emahnuel Troisi Lopez, Arianna Polverino, R.M., Antonella Romano, Viktor Jirsa, Giovanni Rabuffo and Pierpaolo Sorrentino:** visualisation. **Viktor Jirsa, Giovanni Rabuffo and Pierpaolo Sorrentino:** supervision and project administration. **Viktor Jirsa and Pierpaolo Sorrentino:** funding acquisition. All authors read and approved the final manuscript.

## Ethics Statement

The study was approved by the Ethics Committee ASL‐NA1 centro (Prot.n.93C.E./Reg. n.14‐17OSS), and all participants provided written informed consent.

## Conflicts of Interest

The authors declare no conflicts of interest.

### Peer Review

The peer review history for this article is available at https://www.webofscience.com/api/gateway/wos/peer‐review/10.1111/ejn.70128.

## Supporting information


**Figure S1.** Analysis of the entropy of our method output with various thresholds for normalised data (top), and average of the maximum correlations between the default cluster patterns and the patterns retrieved at different thresholds (bottom).
**Figure S2.** Explained variance (A), logarithmic variance (B) and cumulative explained variance (C) as a function of the number of components for the PCA.
**Figure S3.** Distance of KNN in PCA for different numbers of components for the different steps of the pipeline.
**Figure S4.** Comparison of numbers of clusters for PHATE and PCA.
**Figure S5.** Significant diagonal under the second null model.
**Figure S6.** Significance of the recruitment/exclusion of each brain region into each cluster.
**Figure S7.** Transition probability matrix for all subjects and for each one.
**Figure S8.** Significant transitions matrices for all subjects and for each one.
**Figure S9.** Cluster comparison between PHATE for each of the 90 brain regions for 18 versus 44 subjects (seven clusters chosen).
**Figure S10.** Comparison of PCA results from the different steps of the pipeline according to time or per subject.
**Figure S11.** PCA on reconstructed z‐scored MEG source signals.
**Figure S12.** PCA on avalanches.
**Figure S13.** PCA on avalanches patterns
**Figure S14.** Comparison of PHATE results from the different steps of the pipeline
**Figure S15.** The cumulative of activation in all avalanche patterns
**Figure S16.** Stability of the clusters
**Figure S17.** Variability of the significant transition between clusters.
**Figure S18.** Comparison of the significant transition between clusters in each subject.
**Figure S19.** Cluster comparison between PHATE and spectral clustering for each of the 90 brain regions on the 18 studied subjects (seven clusters chosen).
**Figure S20.** Entropy of the output of each selected method (PHATE and spectral clustering for different numbers of clusters).
**Figure S21.** Entropy of the output of each selected method (PHATE, PCA and spectral clustering) applied on different levels of data processing, for seven clusters.
**Figure S22.** Average of maximum correlations matrix with PHATE, spectral clustering and PCA algorithms on 18 subjects (seven clusters chosen).
**Table S1.** List of the 90 brain regions: 78 cortical regions (yellow) and 12 subcortical regions (red).
**Table S2.** Entropy of the output of each selected method (PHATE, PCA and spectral clustering) applied on different levels of data processing, for seven clusters.
**Table S3.** Entropy of the output of each selected method (PHATE and spectral clustering for different numbers of clusters).
**Table S4.** Average of maximum correlations between PHATE and different algorithms (PHATE, spectral clustering and PCA).

## Data Availability

The data cannot be made public due to its personal nature, and as this was foreseen in the informed consent. The code is openly and publicly accessible at: https://github.com/lionelkusch/patien‐analysis.
