# Supplementary material for: Magnetoencephalography Dimensionality Reduction Informed by Dynamic Brain States
Source: Eur J Neurosci. 2025 May 12;61(9):e70128. doi: 10.1111/ejn.70128 (PMC12067517; doi:10.1111/ejn.70128)
Supplement: Supplementary file 1 — Figure S1. Analysis of the entropy of our method output with various thresholds for normalised data (top), and average of the maximum correlations between the default cluster patterns and the patterns retrieved at different thresholds (bottom). Figure S2. Explained variance (A), logarithmic variance (B) and cumulative explained variance (C) as a function of the number of components for the PCA. Figure S3. Distance of KNN in PCA for different numbers of components for the different steps of the pipeline. Figure S4. Comparison of numbers of clusters for PHATE and PCA. Figure S5. Significant diagonal under the second null model. Figure S6. Significance of the recruitment/exclusion of each brain region into each cluster. Figure S7. Transition probability matrix for all subjects and for each one. Figure S8. Significant transitions matrices for all subjects and for each one. Figure S9. Cluster comparison between PHATE for each of the 90 brain regions for 18 versus 44 subjects (seven clusters chosen). Figure S10. Comparison of PCA results from the different steps of the pipeline according to time or per subject. Figure S11. PCA on reconstructed z‐scored MEG source signals. Figure S12. PCA on avalanches. Figure S13. PCA on avalanches patterns Figure S14. Comparison of PHATE results from the different steps of the pipeline Figure S15. The cumulative of activation in all avalanche patterns Figure S16. Stability of the clusters Figure S17. Variability of the significant transition between clusters. Figure S18. Comparison of the significant transition between clusters in each subject. Figure S19. Cluster comparison between PHATE and spectral clustering for each of the 90 brain regions on the 18 studied subjects (seven clusters chosen). Figure S20. Entropy of the output of each selected method (PHATE and spectral clustering for different numbers of clusters). Figure S21. Entropy of the output of each selected method (PHATE, PCA and spectral clustering) applied on different level [file EJN-61-0-s001.docx]

# Supplementary figures

**
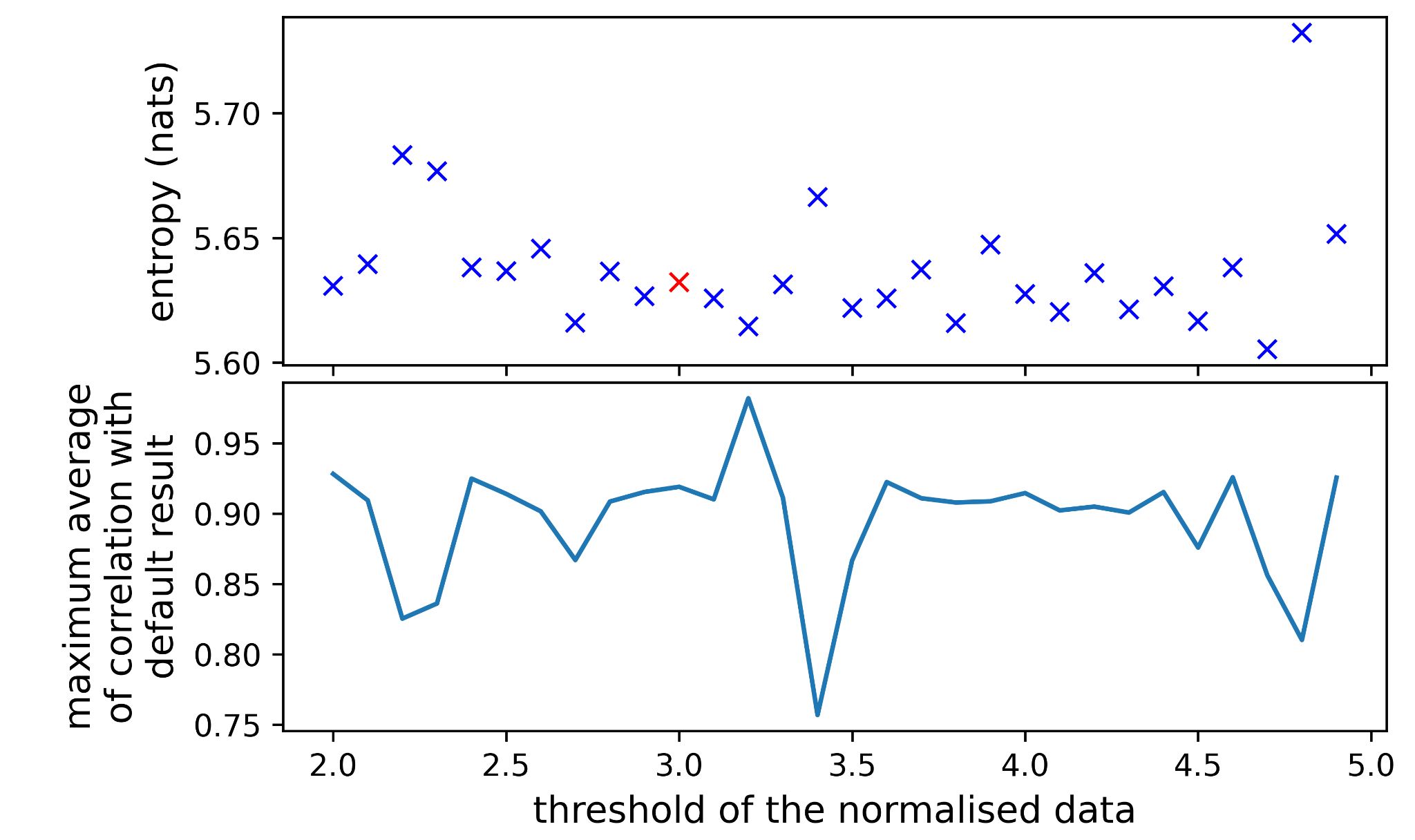
**

## Figure SP1 . Analysis of the entropy of our method output with various thresholds for normalised data (top), and average of the maximum correlations between the default cluster patterns and the patterns retrieved at different thresholds (bottom)

The reliability of clustering across varying z-thresholds is shown by representing the entropy of different thresholds (top panel) and the average of the maximum correlation between results obtained with different thresholds (bottom panel) as a function of the z-score. High correlation values and stable entropy measures indicate that the clustering results remain consistent across different threshold choices.


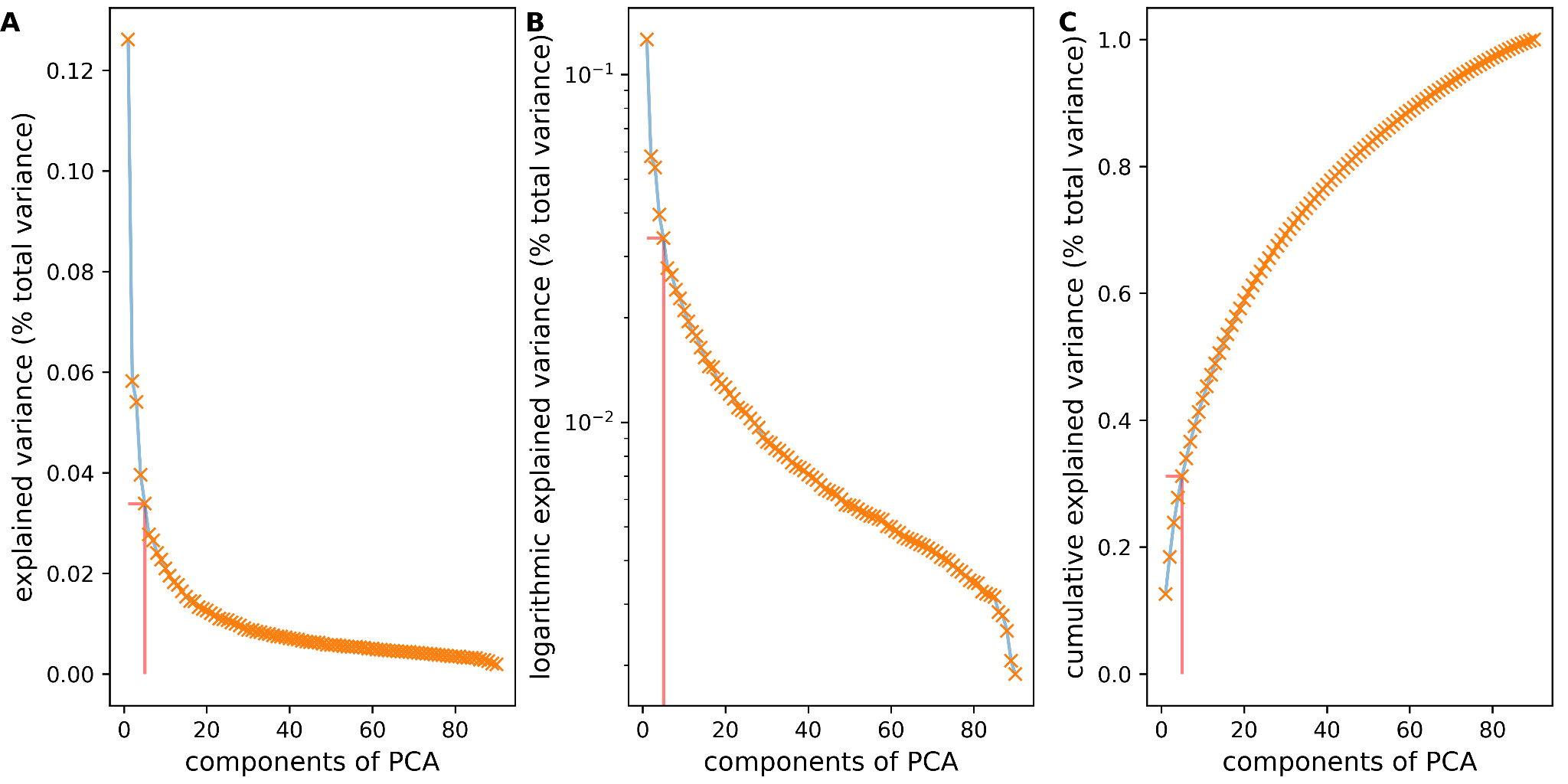


## Figure SP2 . Explained variance (A), logarithmic variance (B), and cumulative explained variance (C) as a function of the number of components for the PCA

This figure displays PCA explained variance (as a percentage of the total variance) by component (left and centre) and PCA cumulative explain variance (right).


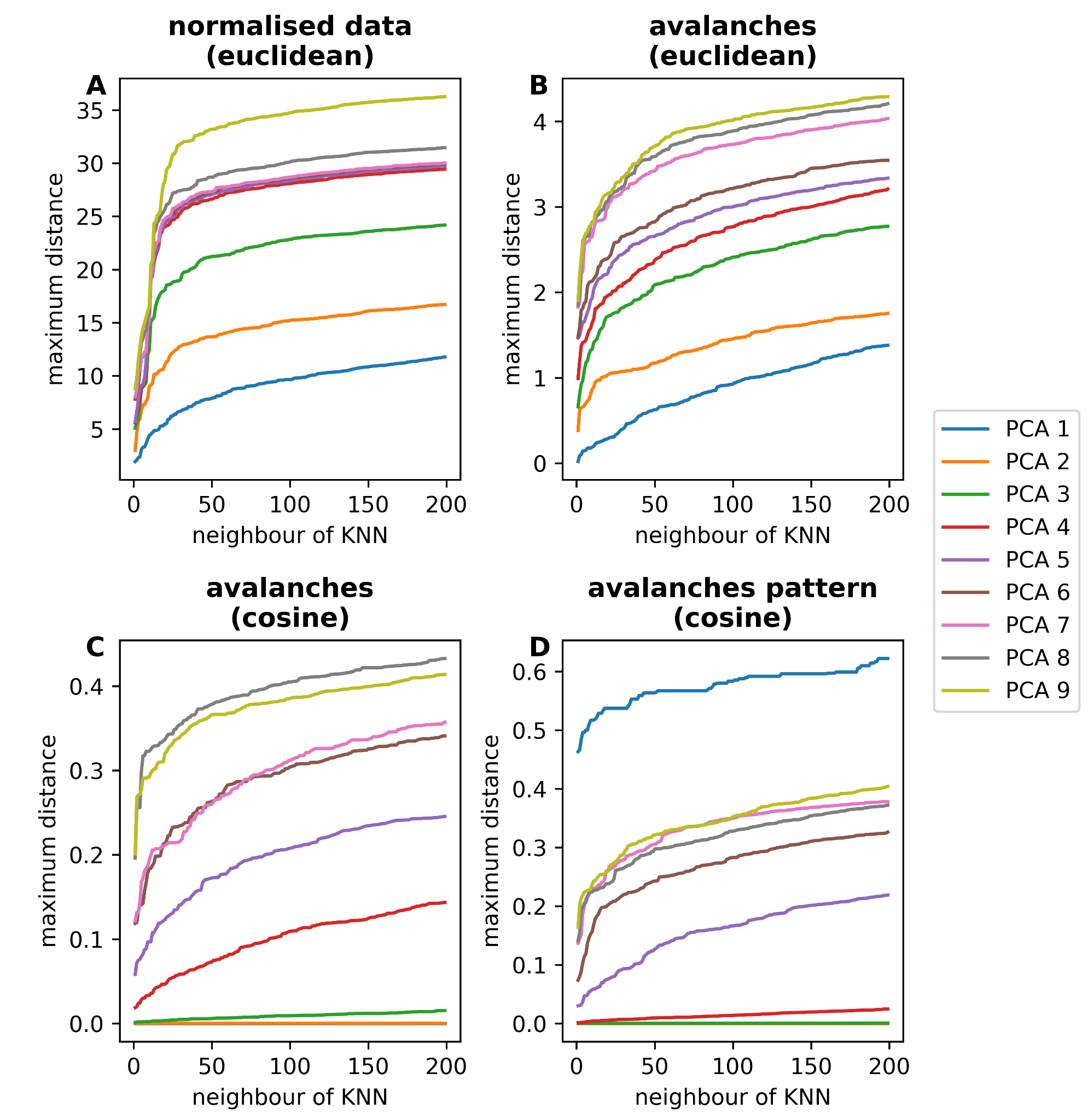


## Figure SP3 . Distance of KNN in PCA for different numbers of components for the different steps of the pipeline

The maximal distance between k-neighbours in KNN is evaluated based on euclidean distance for normalised data (**A**) and avalanches (**B**) and on cosine distance for avalanches (**C**) and avalanches pattern (**D**). Each line corresponds to a number of PCA components (from 1 to 9).


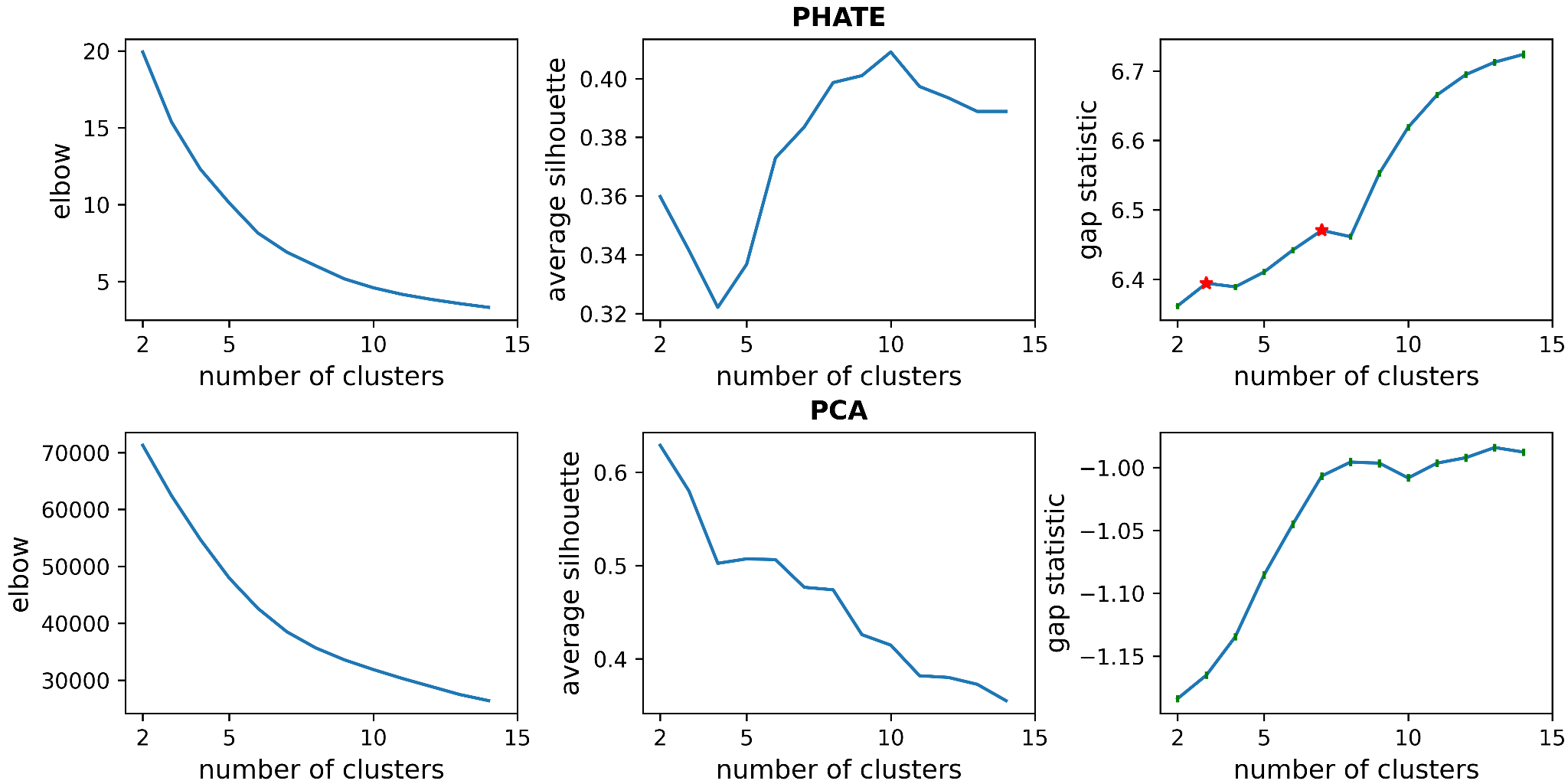


## Figure SP4 . Comparison of numbers of clusters for PHATE and PCA

The top and bottom panels respectively evaluate the number of clusters of low dimension of PHATE and PCA.


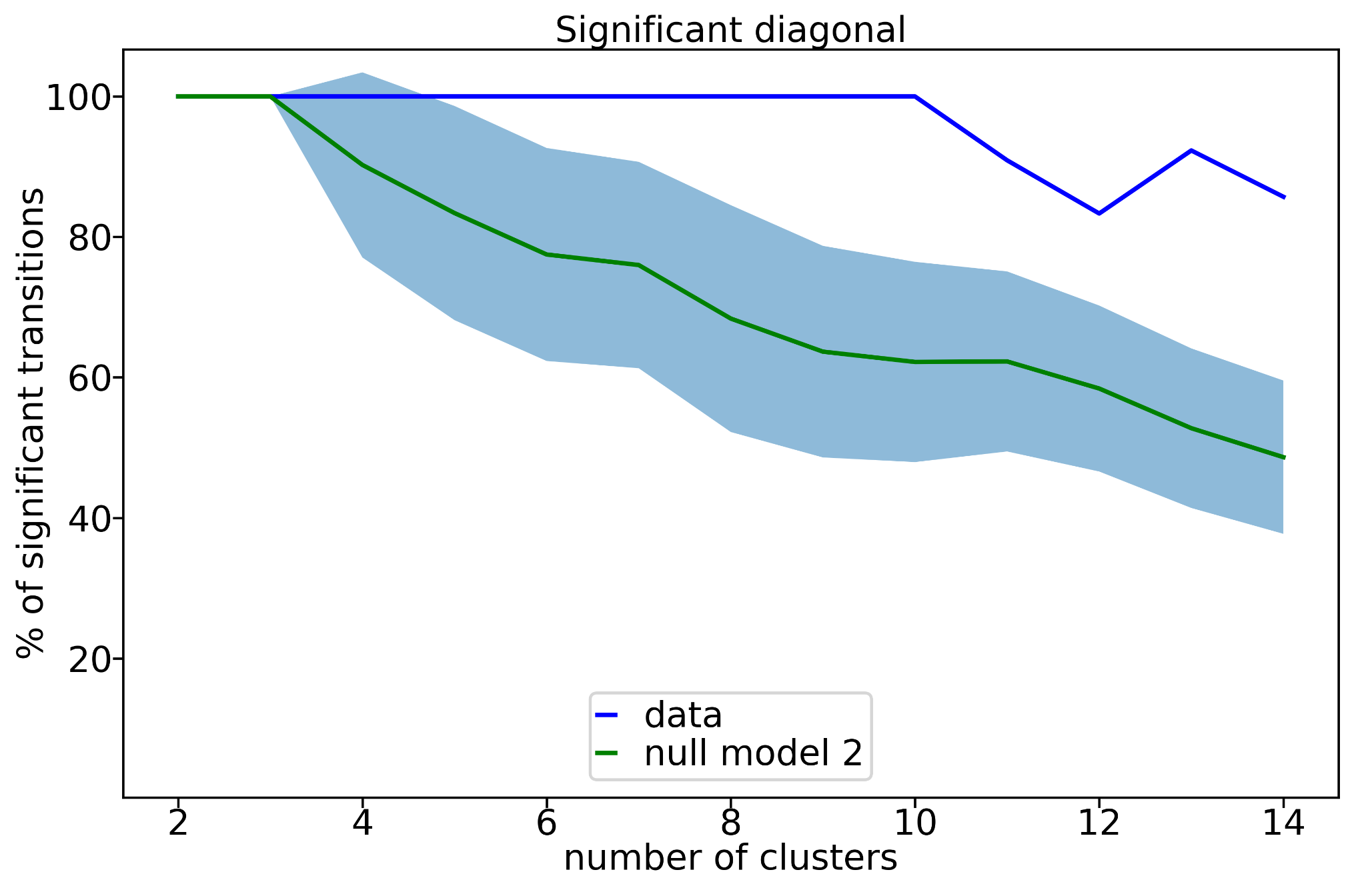


## Figure SP5 . Significant Diagonal under the second null model

The figure presents the distribution (dark turquoise) and mean (green) of the percentages of statistically significant transitions under the second null model, where cluster labels were shuffled while maintaining the proportion of clusters. At each iteration, transition probabilities were recalculated based on the shuffled sequence, generating a null distribution for each cluster. A cutoff of 0.05 was used to define significant transitions. The percentage of significant transitions (over the total number of transitions) in the empirical data is shown in blue. The analysis was repeated for different numbers of clusters, represented along the x-axis.

##
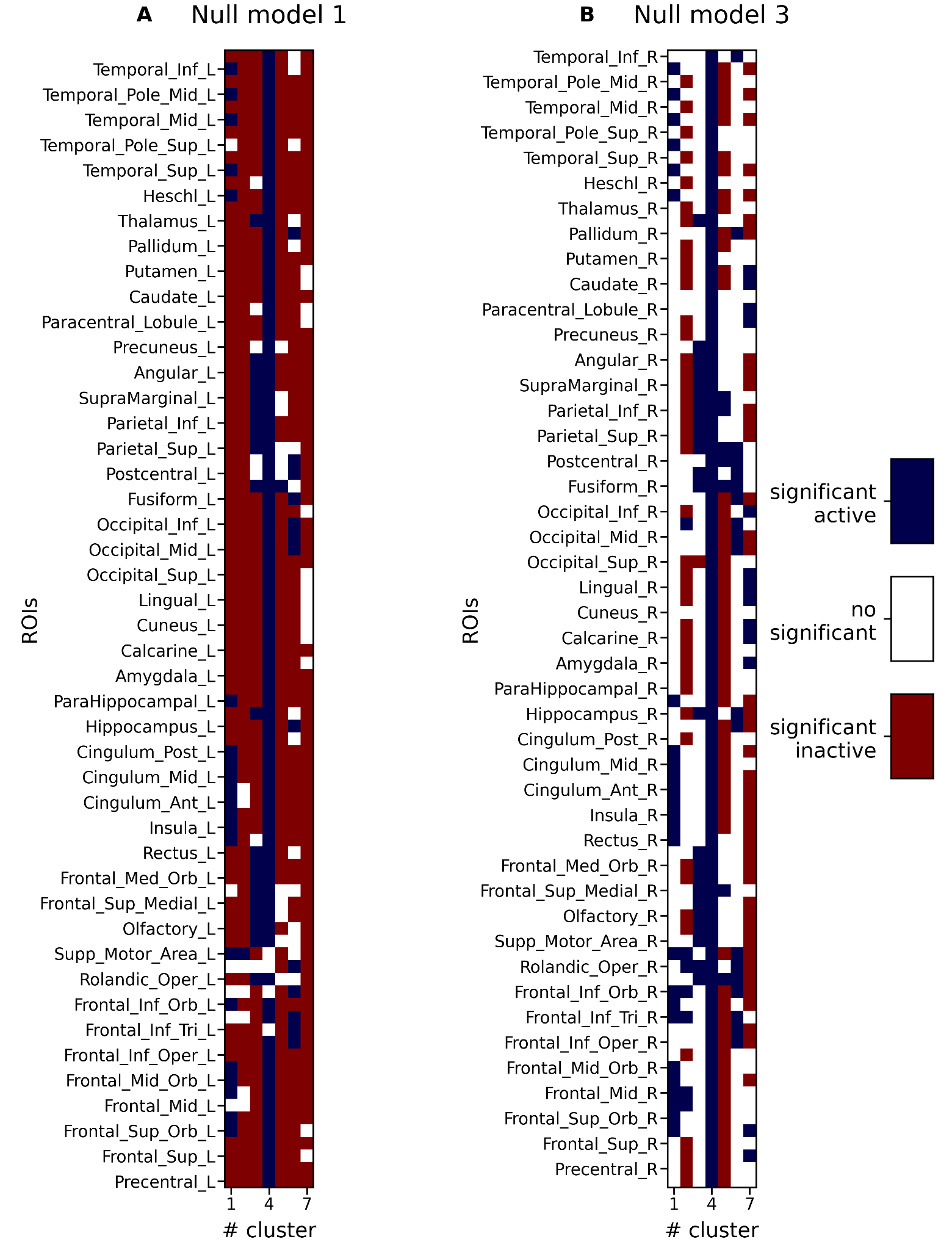


## Figure SP6 . Significance of the recruitment/exclusion of each brain region into each cluster

Each matrix from **A** and **B** reports the significance of the recruitment (above or below chance level) of each cluster for every region. In other words, they represent the sum, region-wise, of the avalanches belonging to each cluster. More specifically, those panels show the significance of the probabilities of a region to be recruited above chance (blue), excluded above chance (red) or not significantly recruited nor excluded (white) for a specific cluster, for a precision at 0.05 (**A**) and 0.2 (**B**).


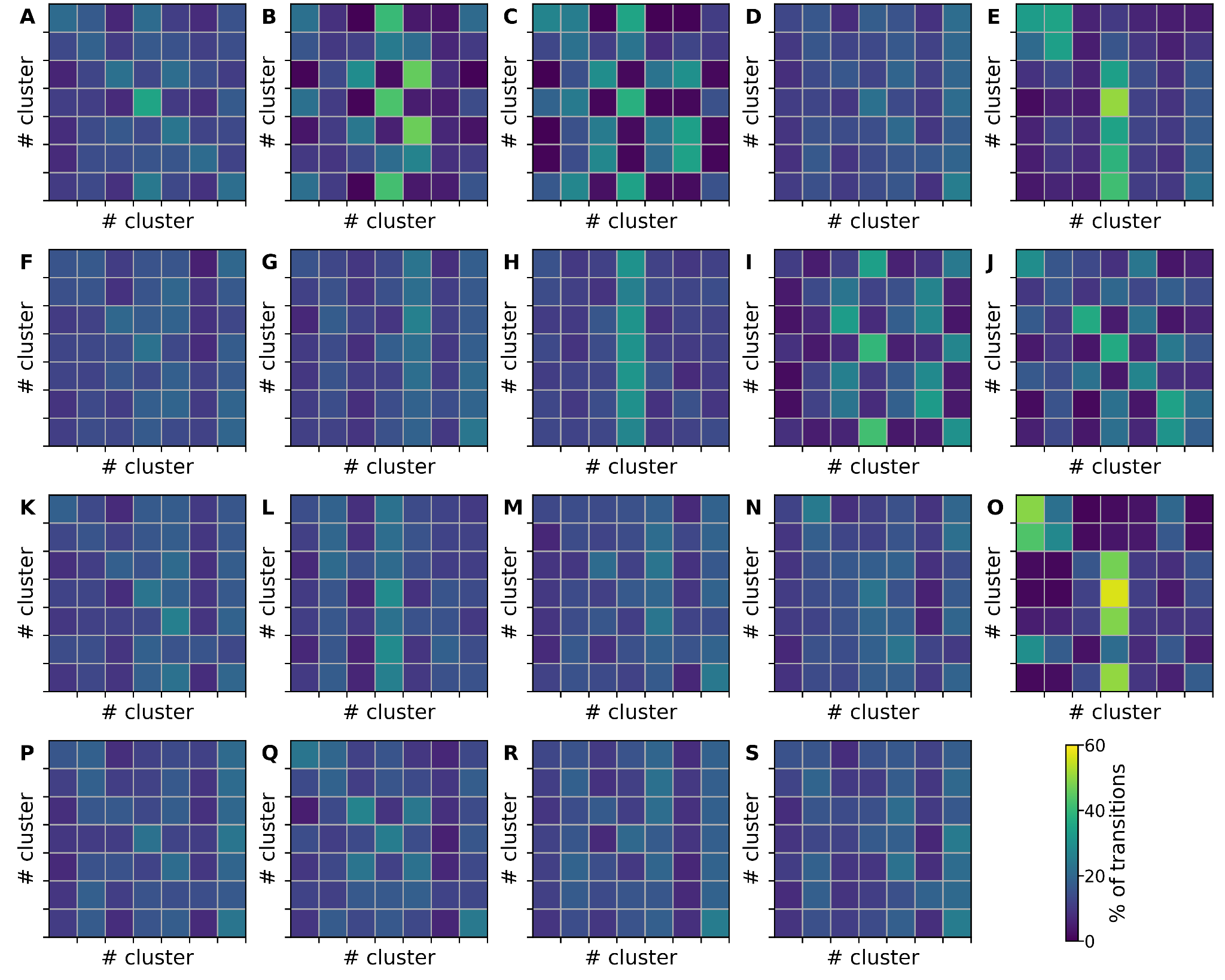


## Figure SP7 . Transition probability matrix for all subjects and for each one

The top left panel (**A)** displays the transition probabilities matrix for all subjects concatenated. The others (**B-S**) represent the transition probabilities matrices for each subject.


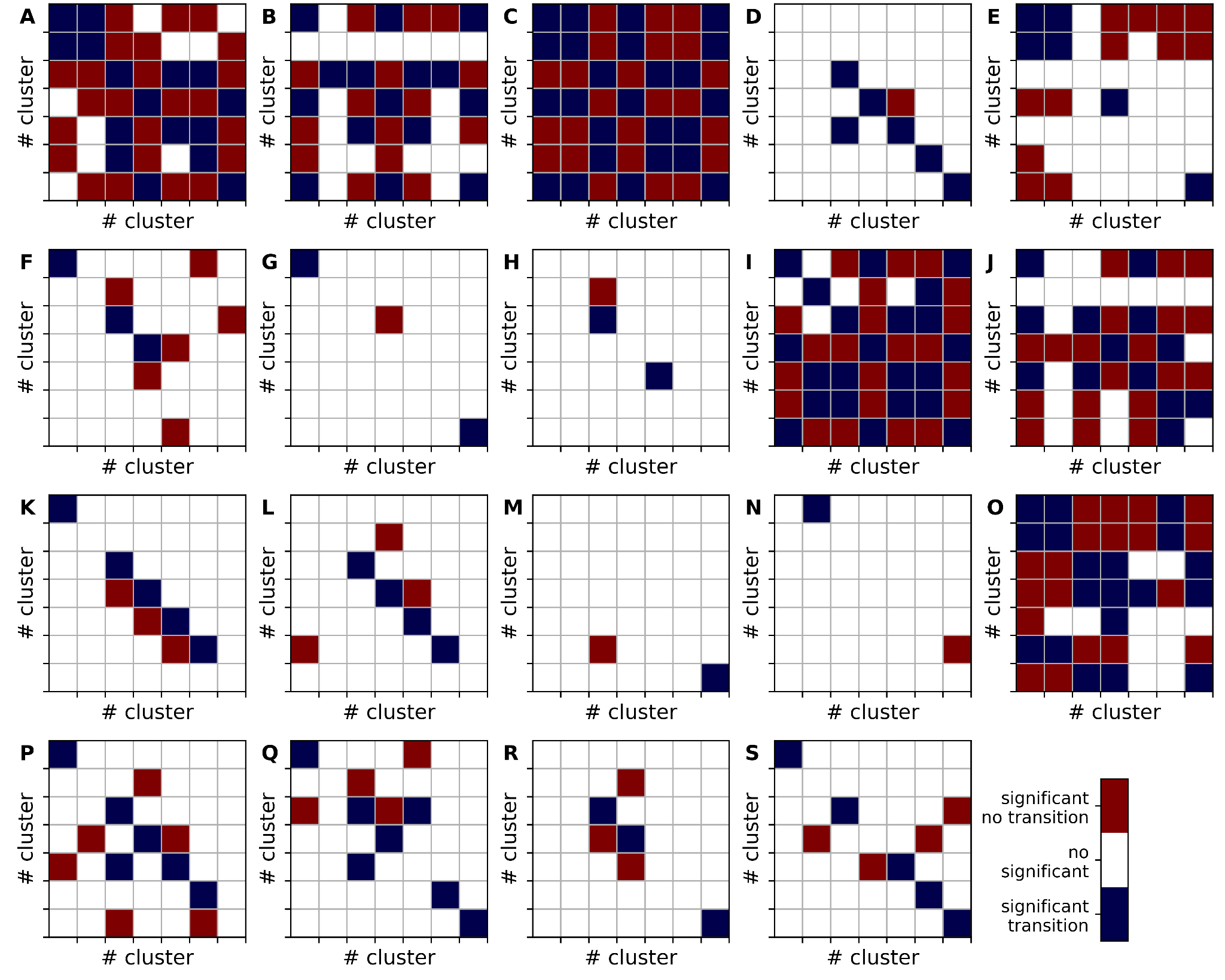


## Figure SP8 . Significant transitions matrices for all subjects and for each one

The top left panel (**A)** represents the significant transitions for all subjects concatenated. The others (**B-S**) represent the significant transitions for each subject. The precision is set at 0.05.


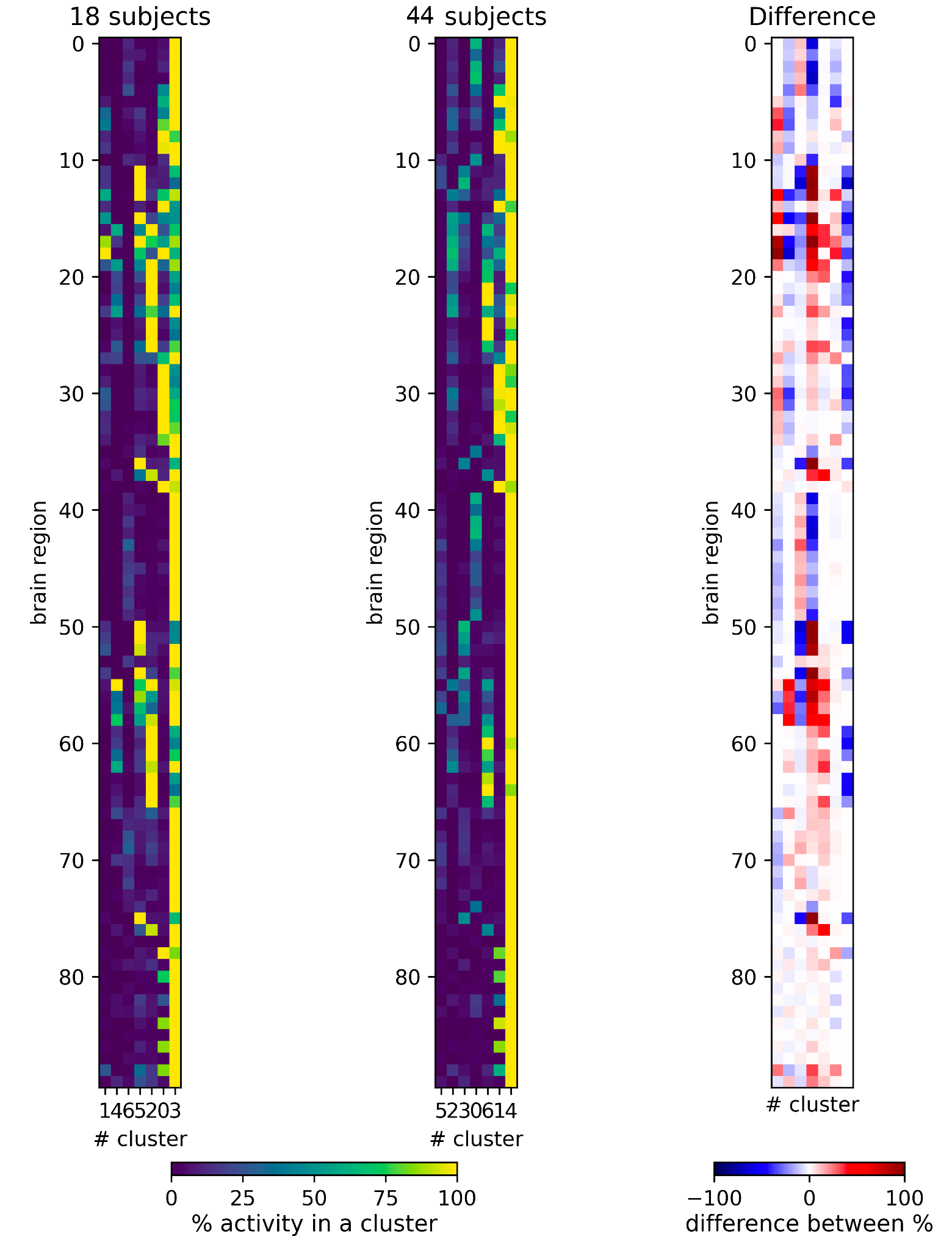


## Figure SP9 . Cluster comparison between PHATE for each of the 90 brain regions for 18 versus 44 subjects. (7 clusters chosen)

Brain clusters defined on the 18-subjects-cohort (left) and the second cohort of 44 subjects (center). The corresponding differences are shown to the right.


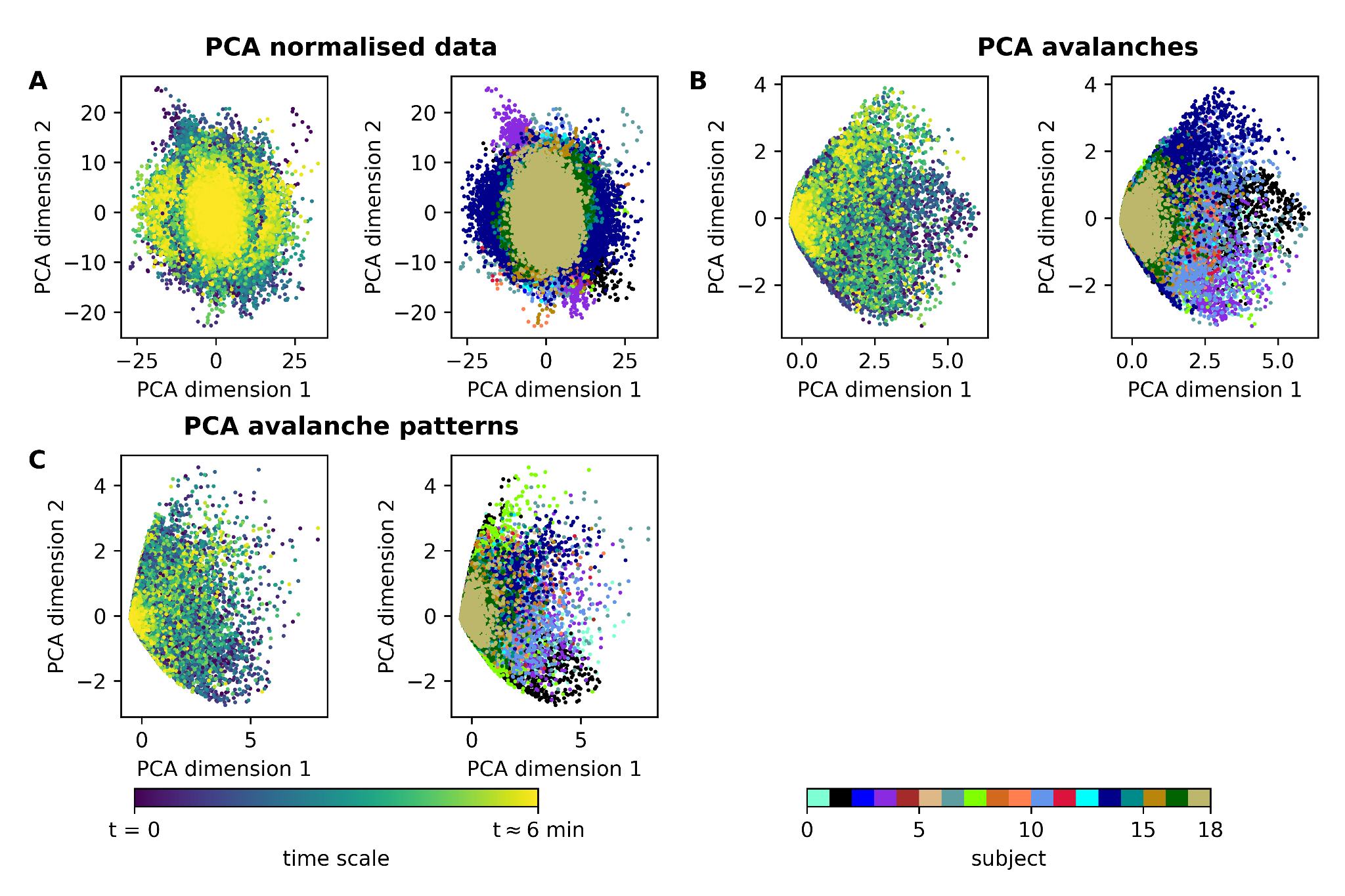


## Figure SP10 . Comparison of PCA results from the different steps of the pipeline according to time or per subject

The results of PCA for the different steps of the pipeline (**A**: reconstructed z-scored Meg source signal, **B**: binarized activity and **C**: avalanches pattern) compares the time evolution of brain state (left, time is the colour code) and subject data (right, subject is the colour code) for the subjects in the two first components of the PCA space. It is to be noted that the duration (t end, around 6 min) varies for each subplot, as different subjects exhibit different durations.

##
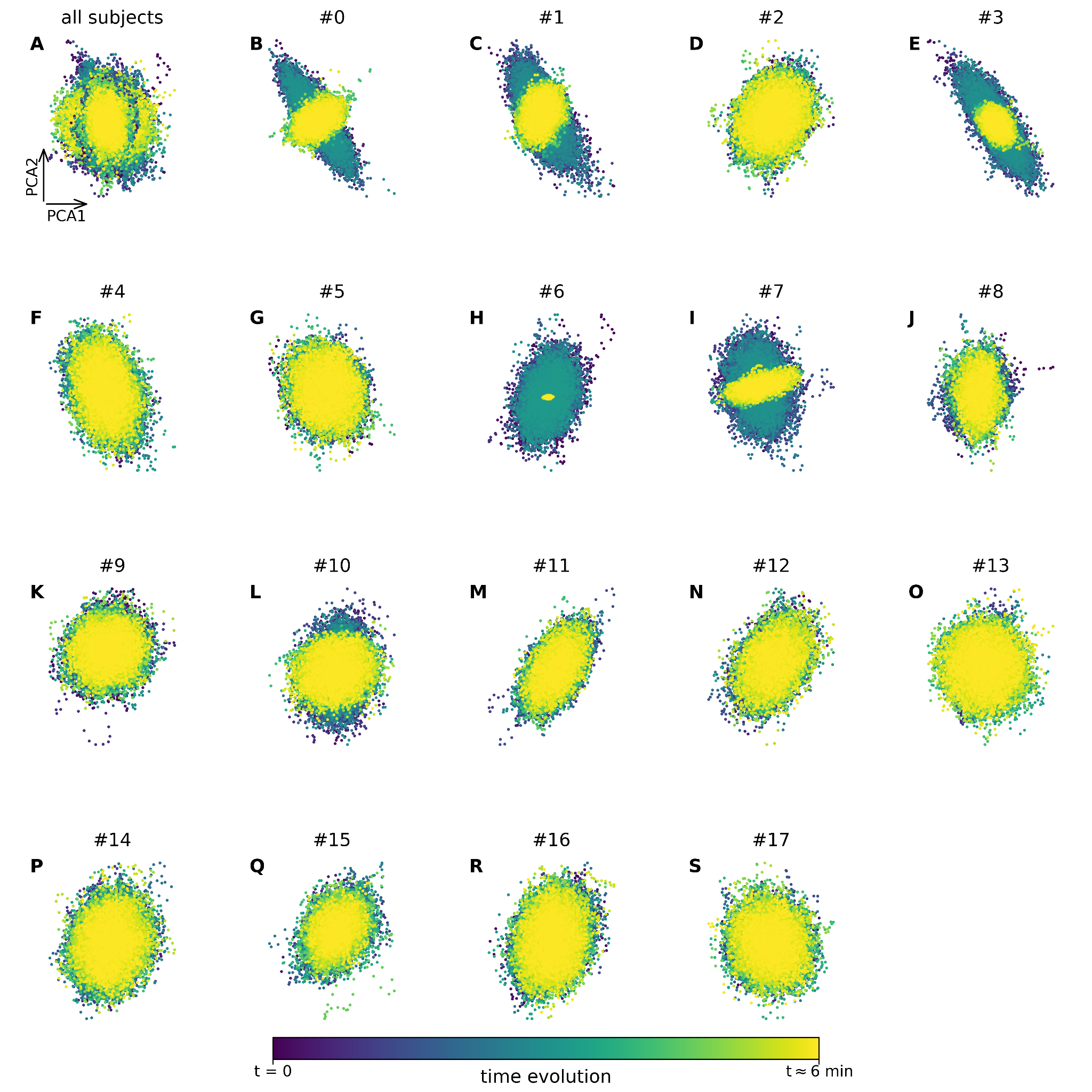


## Figure SP11 . PCA on reconstructed z-scored MEG source signals

The graphic shows the time evolution of brain state for the subjects in the two first components of the PCA space. The top left panel (**A**) corresponds to a superposition of all subjects. The other graphs (panels **B-S**) are for each subject. Hence, in these plots, each dot, each dot represents the evolution of the whole system brain state at each instant (colour code).


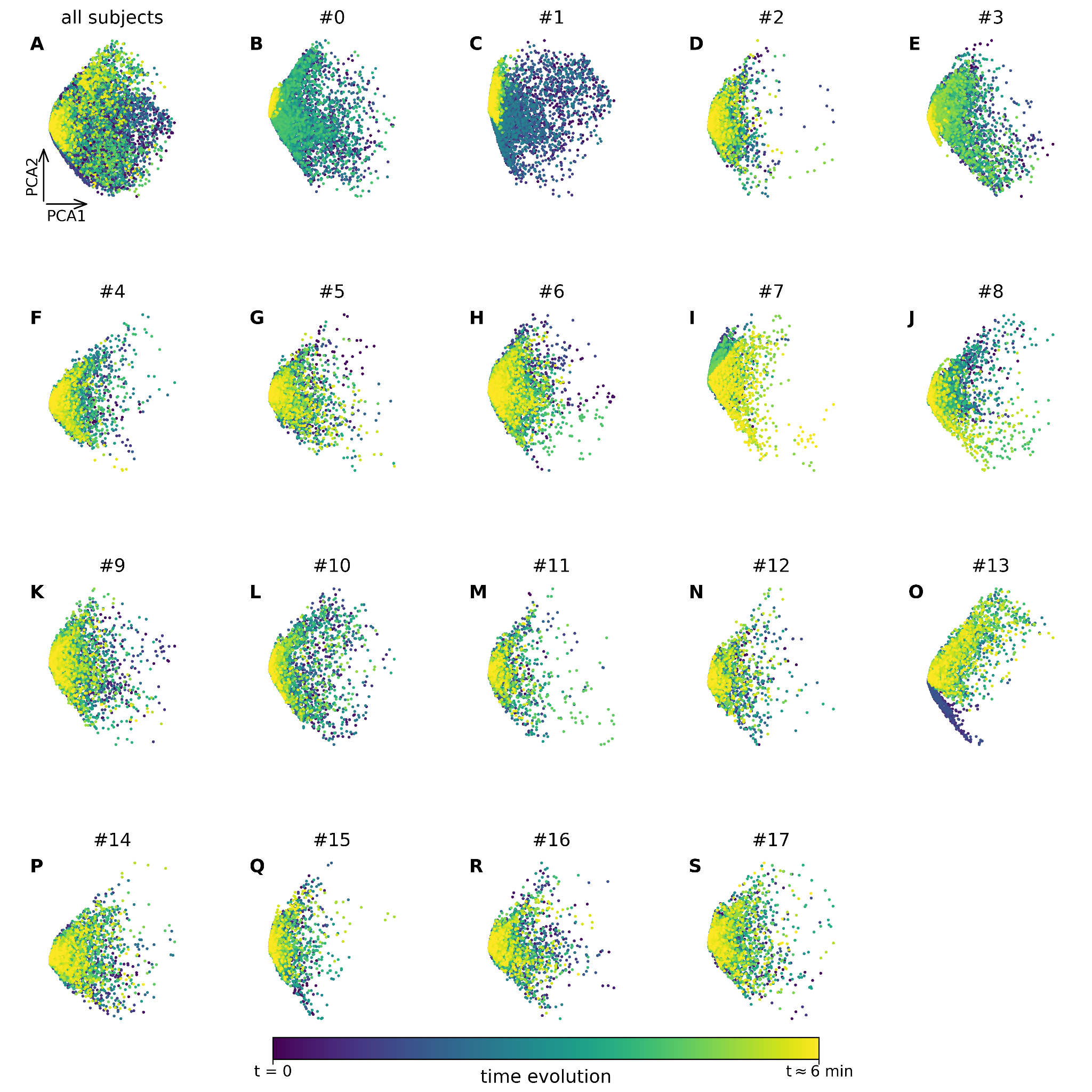


## Figure SP12 . PCA on avalanches

The graphic shows the time evolution of brain state for the subjects in the two first components of the PCA space. The top left panel (**A**) corresponds to a superposition of all subjects. The other graphs (panels **B-S**) are for each subject. In these plots, each dot, each dot represents the evolution of the whole system brain state at each instant (colour code). The duration (t end, around 6 min) varies for each subplot, as different subjects exhibit different durations.


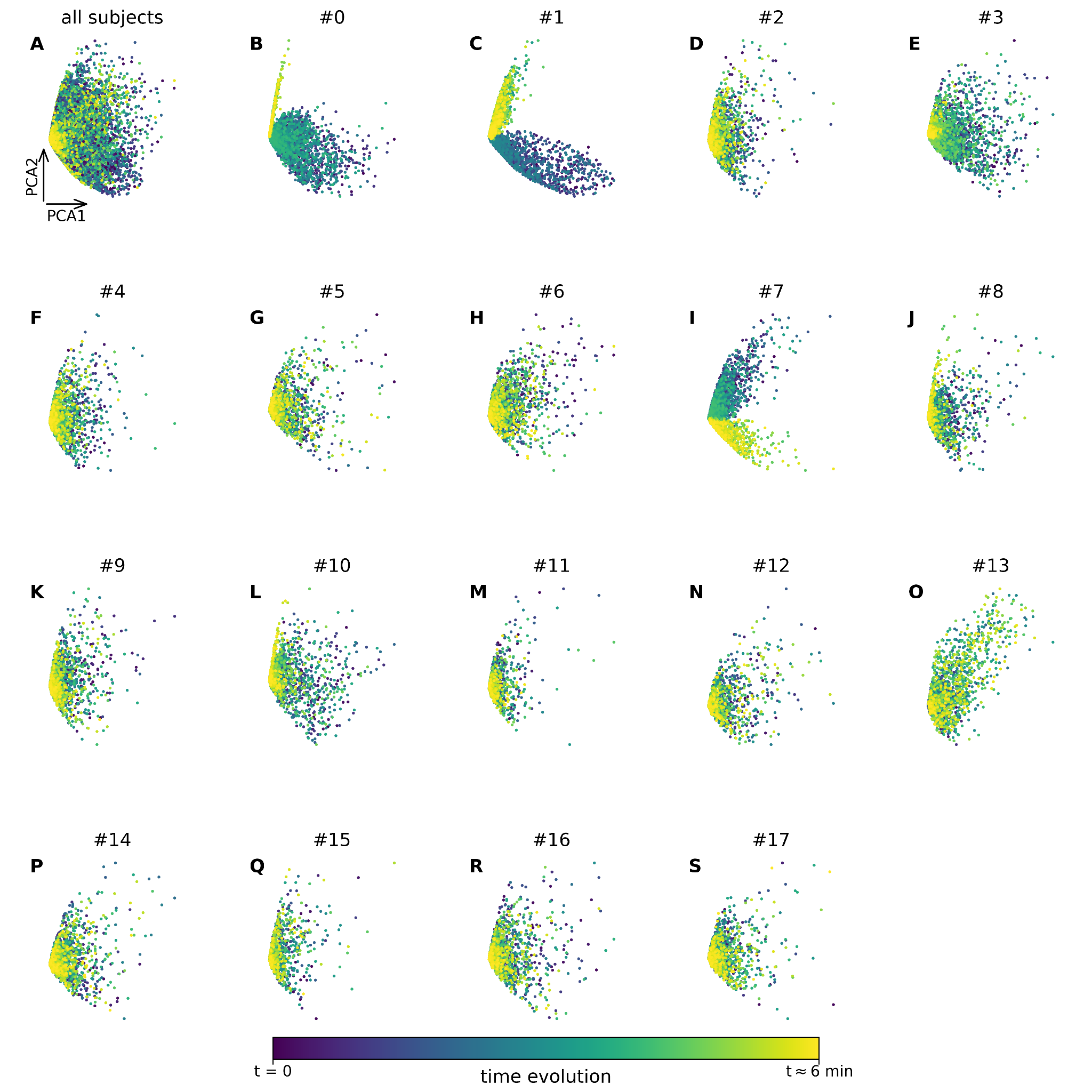


## Figure SP13 . PCA on avalanches patterns

The graphic shows the time evolution of brain state for the subjects in the two first components of the PCA space. The top left panel (**A**) corresponds to a superposition of all subjects. The other graphs (panels **B-S**) are for each subject. In these plots, each dot, each dot represents the evolution of the whole system brain state at each instant (colour code). The duration (t end, around 6 min) varies for each subplot, as different subjects exhibit different durations.

##
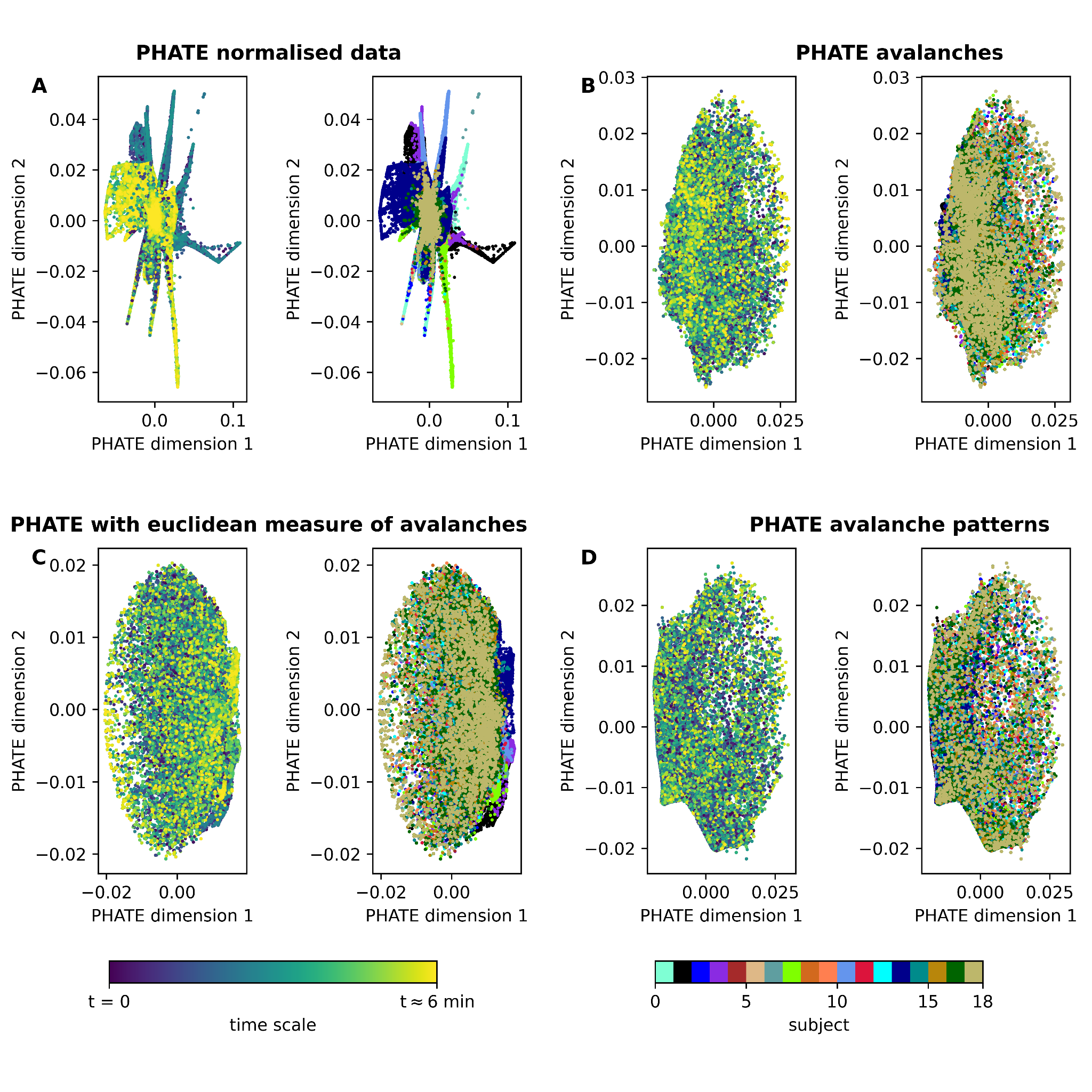


## Figure SP14 . Comparison of PHATE results from the different steps of the pipeline

The result of PHATE for the different steps of the pipeline (**A**: z-scored signal, **B**,**C**: binarized activity and **C**: avalanches pattern) shows the time evolution of brain state (left, time is the colour code) and subject data (right, subject is the colour code) for the subjects in the two first components of the PHATE space. It should be considered that the duration (t end, around 6 min) varies for each subplot, as different subjects exhibit different durations.

##

##
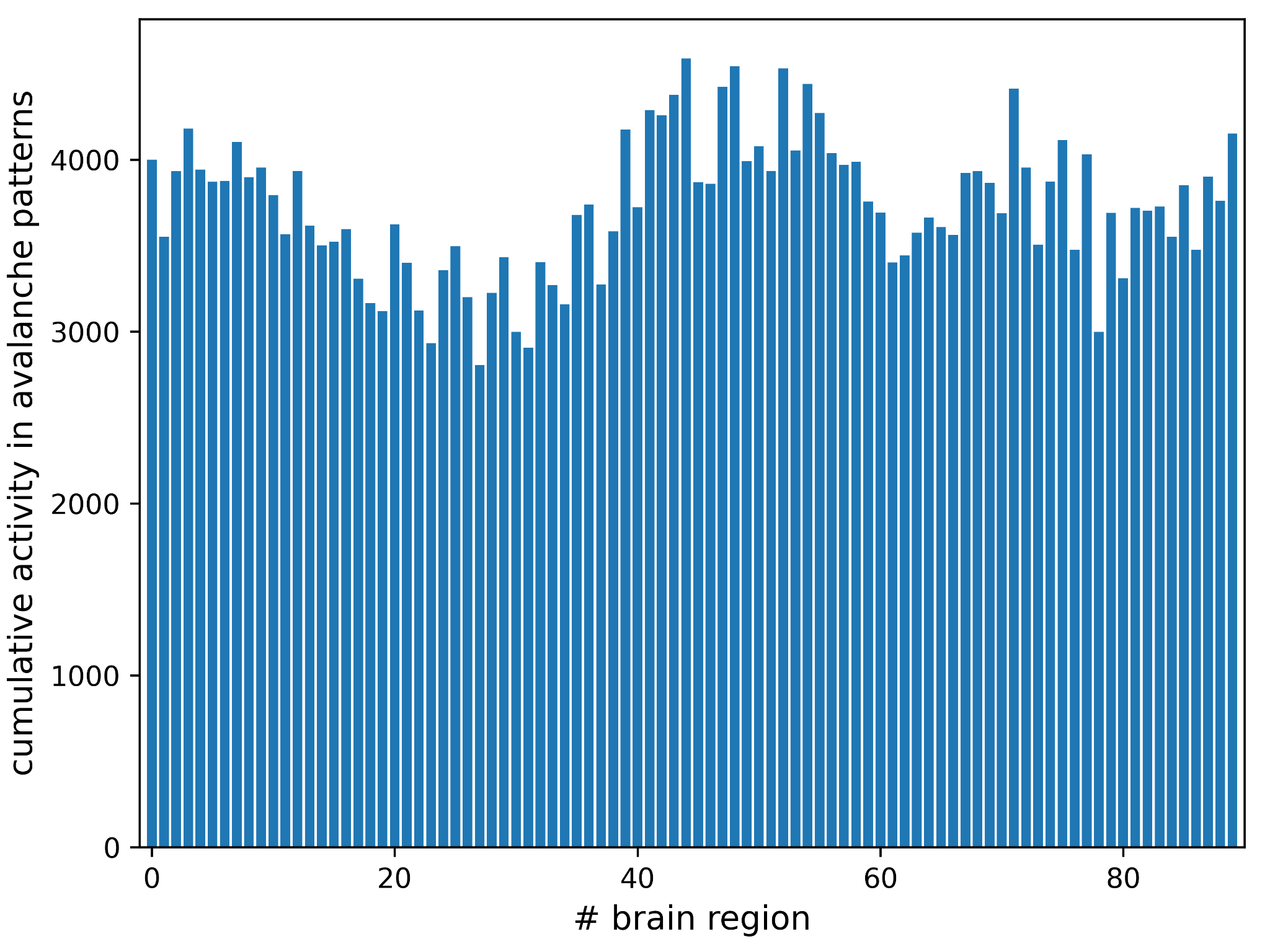


## Figure SP15 . The cumulative of activation in all avalanche patterns

The figure represents the cumulative activation of each region in avalanches pattern.

##
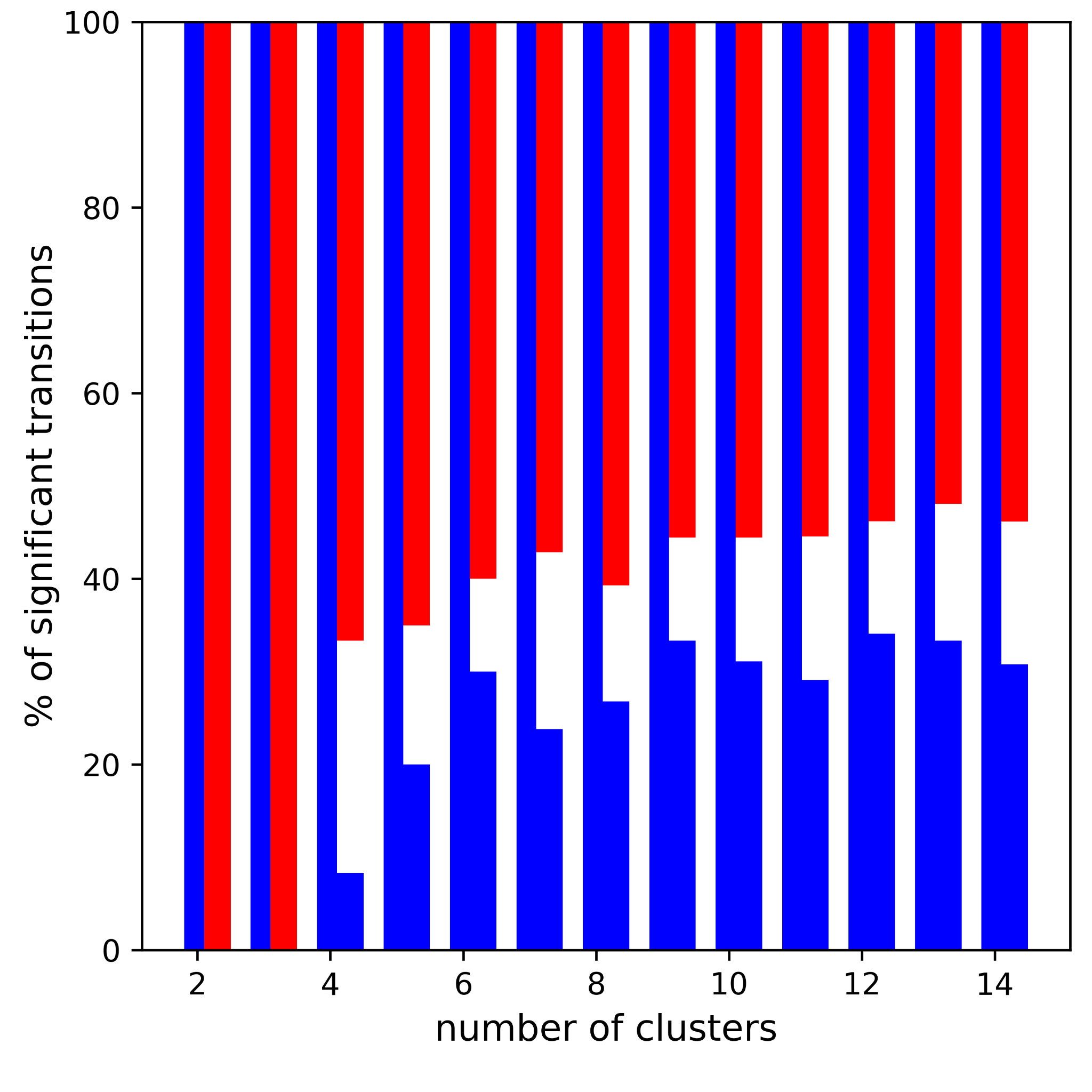


## Figure SP16 . Stability of the clusters

This panel displays two columns by different numbers of clusters. The left column corresponds to the percentage of the diagonal matrix of the transition between clusters that is higher or lower than chance and the right column is for the rest of the transition. The blue indicates the percentage of transitions which are lower than chance and the red, the percentage of transitions which are higher than chance. The significance threshold is set at 0.05.


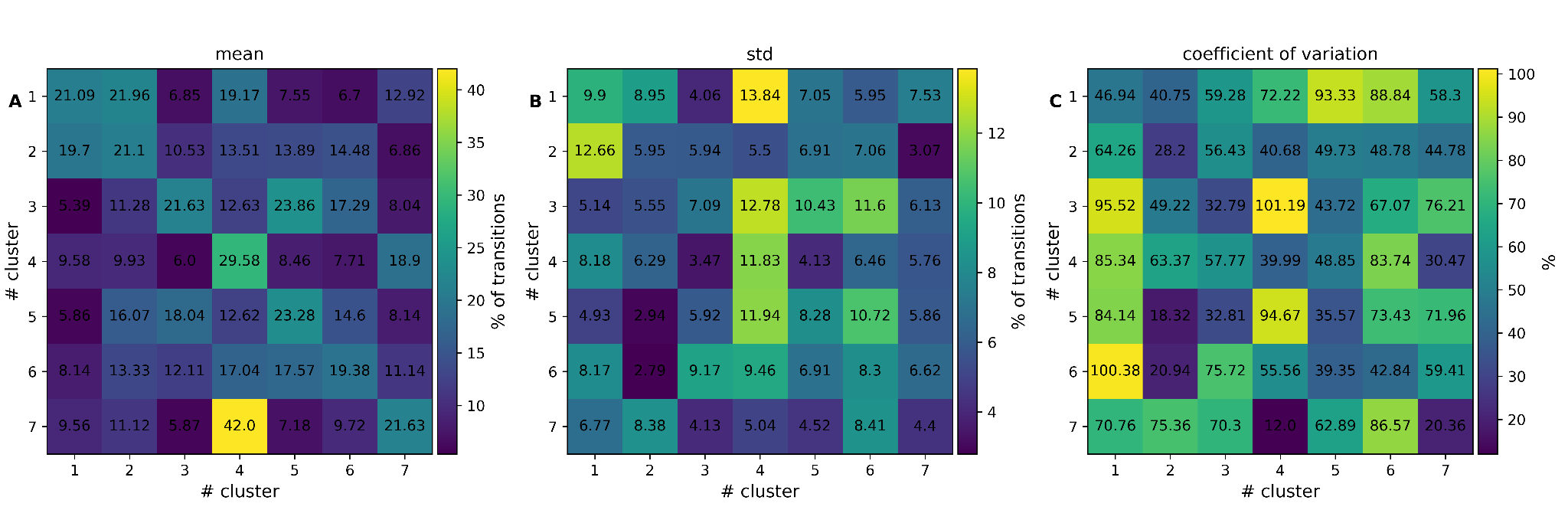


## Figure SP17 . Variability of the significant transition between clusters

The graphics represent the mean (**A**), standard deviation (**B**) and the coefficient of variation (**C**) of the transition of all subjects.

##
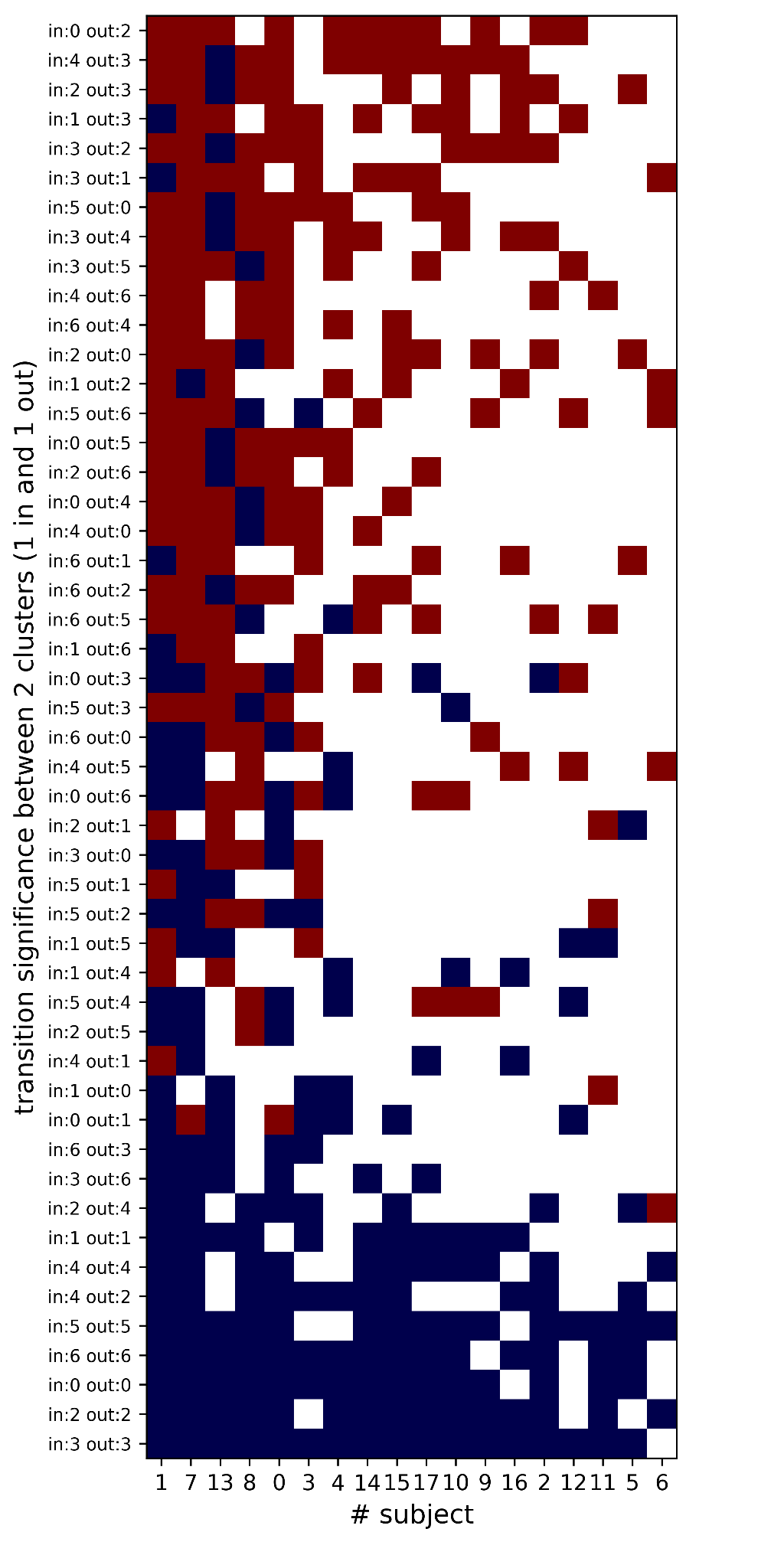


## Figure SP18 . Comparison of the significant transition between cluster in each subject

The transitions between clusters are ordered starting from the cluster with the highest transition probability. The subjects are ordered as a function of the number of significant transitions. One can see the significances of the probabilities of a region being recruited above chance (in blue), excluded above chance (in red), or not being significantly recruited/excluded in a cluster (above chance - in white), at a significance level of 0.05


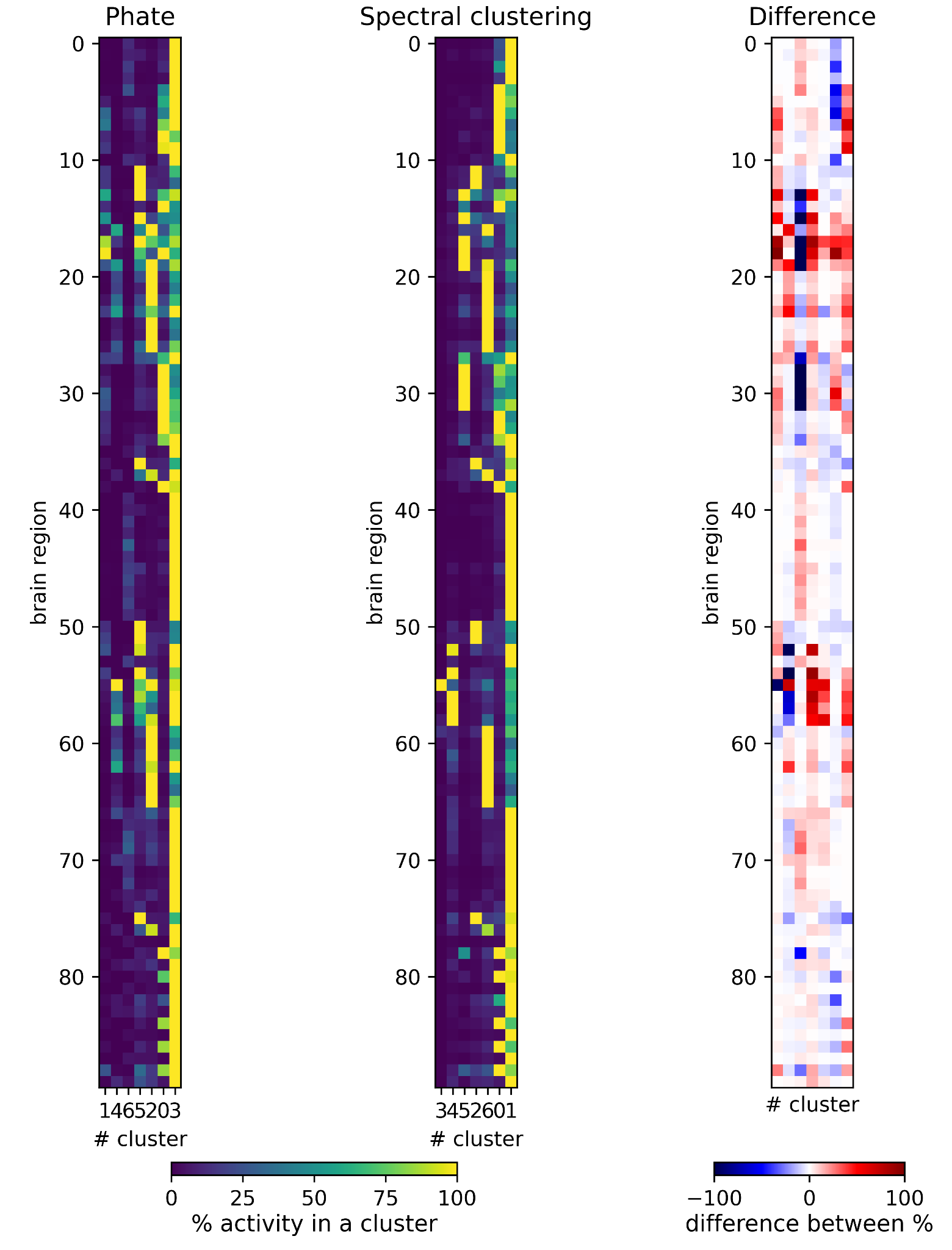


## Figure SP19 . Cluster comparison between PHATE and Spectral clustering for each of the 90 brain regions on the 18 studied subjects. (7 clusters chosen)

The comparison of clustering results between the PHATE (left) and Spectral clustering (center) algorithms is made possible by the display of clusters for both cohorts alongside their corresponding differences (right). All algorithms are applied on the data of the 18 subjects studied and the number of output clusters is fixed at 7.


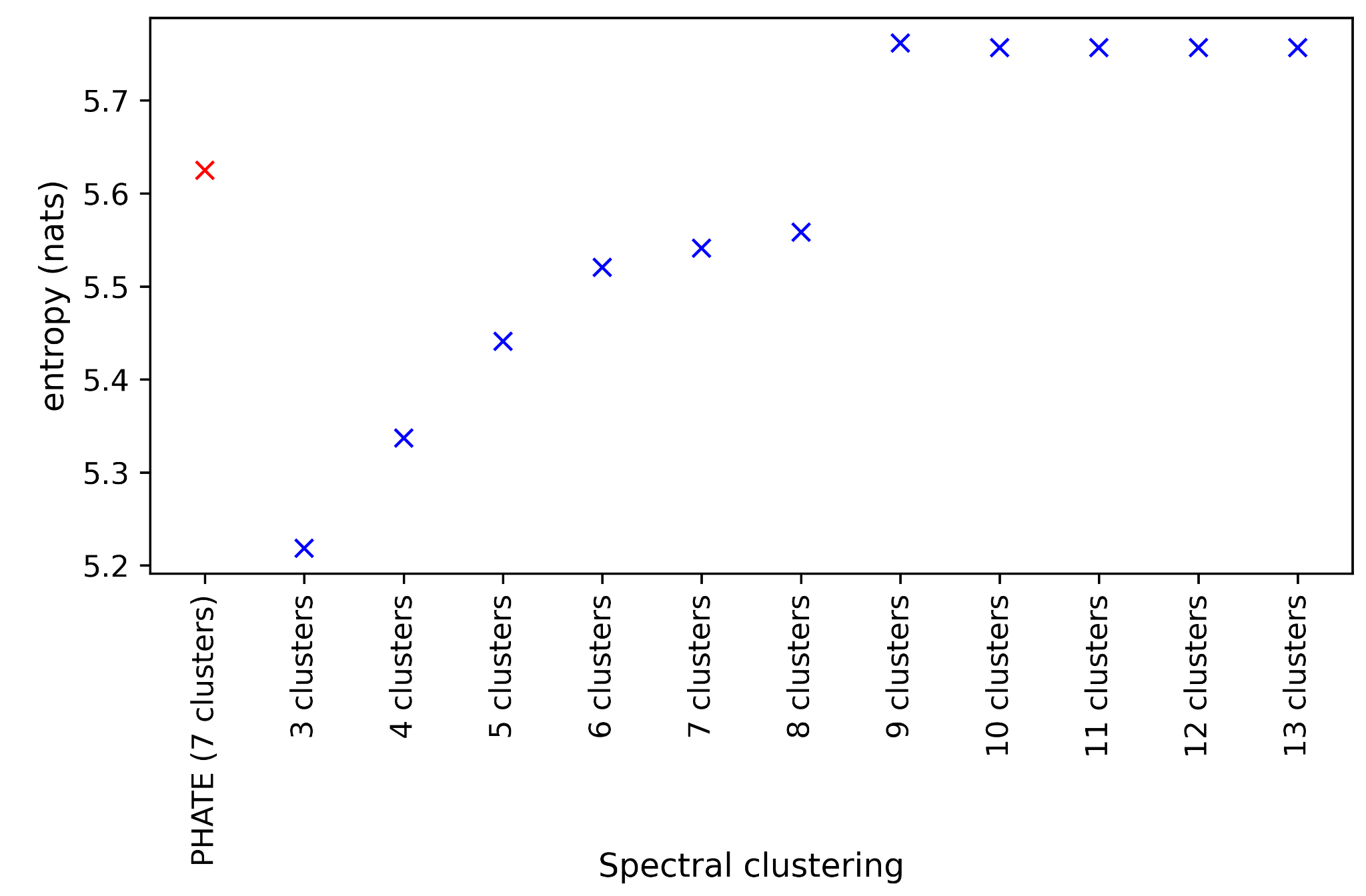


## Figure SP20 . Entropy of the output of each selected method (PHATE and Spectral clustering for different numbers of clusters)

##
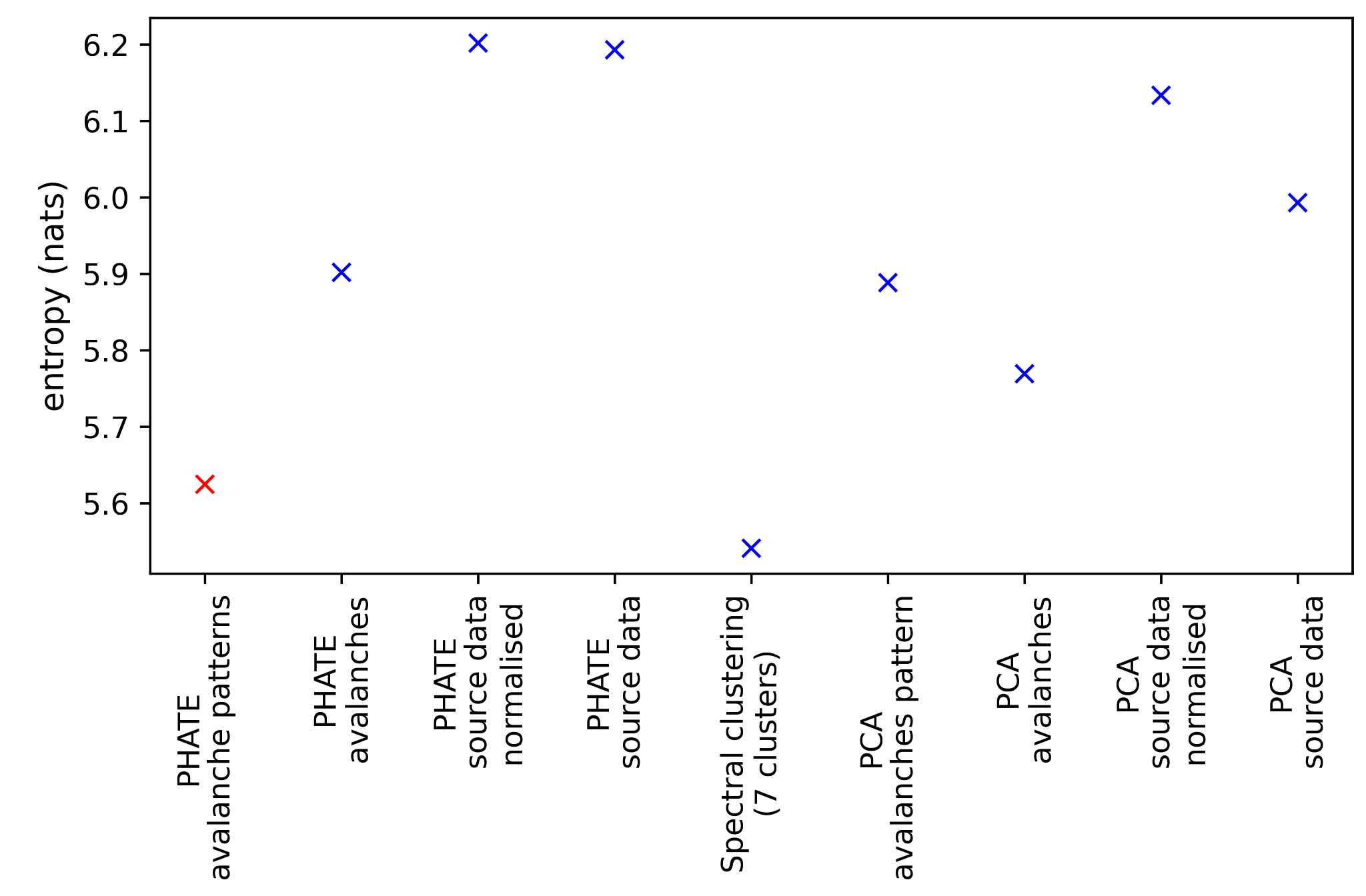


## Figure SP21 . Entropy of the output of each selected method (PHATE, PCA and Spectral clustering) applied on different levels of data processing, for 7 clusters


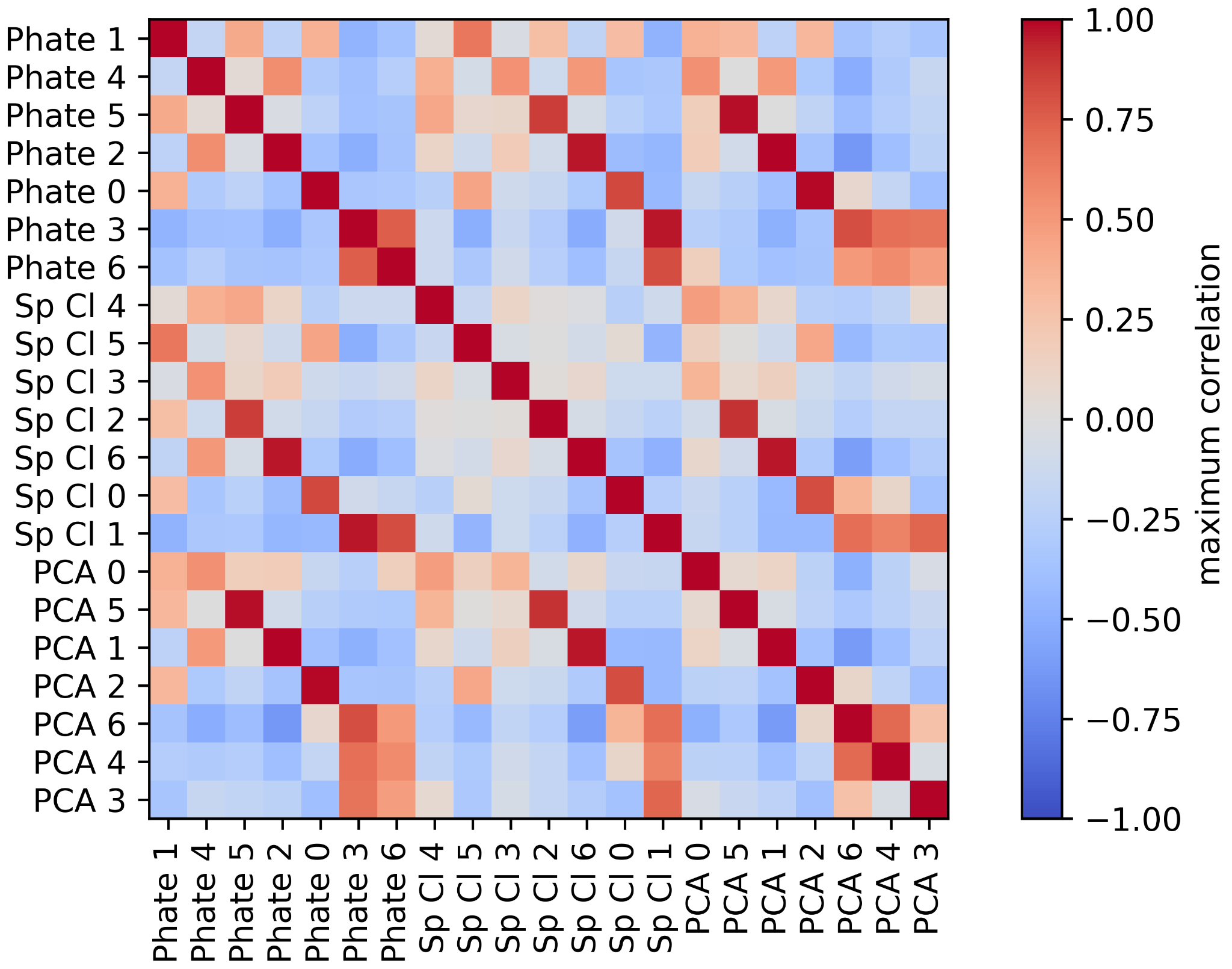


## Figure SP22 . Average of maximum correlations matrix with PHATE, Spectral clustering and PCA algorithms on 18 subjects (7 clusters chosen)

Comparison of cluster similarities across PHATE, PCA, and Spectral clustering using correlation analysis. The correlation values between the topographies of the seven clusters obtained by each method are displayed in the matrix. Higher correlation values indicate stronger similarity between clusters across methods.

# Supplementary tables

## Table ST1 . List of the 90 brain regions: 78 cortical regions (yellow) and 12 subcortical regions (red)

|  | **Brain regions** |
| --- | --- |
| 1 | Rectus_L |
| 2 | Olfactory_L |
| 3 | Frontal_Sup_Orb_L |
| 4 | Frontal_Med_Orb_L |
| 5 | Frontal_Mid_Orb_L |
| 6 | Frontal_Inf_Orb_L |
| 7 | Frontal_Sup_L |
| 8 | Frontal_Mid_L |
| 9 | Frontal_Inf_Oper_L |
| 10 | Frontal_Inf_Tri_L |
| 11 | Frontal_Sup_Medial_L |
| 12 | Supp_Motor_Area_L |
| 13 | Paracentral_Lobule_L |
| 14 | Precentral_L |
| 15 | Rolandic_Oper_L |
| 16 | Postcentral_L |
| 17 | Parietal_Sup_L |
| 18 | Parietal_Inf_L |
| 19 | SupraMarginal_L |
| 20 | Angular_L |
| 21 | Precuneus_L |
| 22 | Occipital_Sup_L |
| 23 | Occipital_Mid_L |
| 24 | Occipital_Inf_L |
| 25 | Calcarine_L |
| 26 | Cuneus_L |
| 27 | Lingual_L |
| 28 | Fusiform_L |
| 29 | Heschl_L |
| 30 | Temporal_Sup_L |
| 31 | Temporal_Mid_L |
| 32 | Temporal_Inf_L |
| 33 | Temporal_Pole_Sup_L |
| 34 | Temporal_Pole_Mid_L |
| 35 | ParaHippocampal_L |
| 36 | Cingulum_Ant_L |
| 37 | Cingulum_Mid_L |
| 38 | Cingulum_Post_L |
| 39 | Insula_L |
| 40 | Rectus_R |
| 41 | Olfactory_R |
| 42 | Frontal_Sup_Orb_R |
| 43 | Frontal_Med_Orb_R |
| 44 | Frontal_Mid_Orb_R |
| 45 | Frontal_Inf_Orb_R |
| 46 | Frontal_Sup_R |
| 47 | Frontal_Mid_R |
| 48 | Frontal_Inf_Oper_R |
| 49 | Frontal_Inf_Tri_R |
| 50 | Frontal_Sup_Medial_R |
| 51 | Supp_Motor_Area_R |
| 52 | Paracentral_Lobule_R |
| 53 | Precentral_R |
| 54 | Rolandic_Oper_R |
| 55 | Postcentral_R |
| 56 | Parietal_Sup_R |
| 57 | Parietal_Inf_R |
| 58 | SupraMarginal_R |
| 59 | Angular_R |
| 60 | Precuneus_R |
| 61 | Occipital_Sup_R |
| 62 | Occipital_Mid_R |
| 63 | Occipital_Inf_R |
| 64 | Calcarine_R |
| 65 | Cuneus_R |
| 66 | Lingual_R |
| 67 | Fusiform_R |
| 68 | Heschl_R |
| 69 | Temporal_Sup_R |
| 70 | Temporal_Mid_R |
| 71 | Temporal_Inf_R |
| 72 | Temporal_Pole_Sup_R |
| 73 | Temporal_Pole_Mid_R |
| 74 | ParaHippocampal_R |
| 75 | Cingulum_Ant_R |
| 76 | Cingulum_Mid_R |
| 77 | Cingulum_Post_R |
| 78 | Insula_R |
| 79 | Hippocampus_L |
| 80 | Hippocampus_R |
| 81 | Amygdala_L |
| 82 | Amygdala_R |
| 83 | Caudate_L |
| 84 | Caudate_R |
| 85 | Putamen_L |
| 86 | Putamen_R |
| 87 | Pallidum_L |
| 88 | Pallidum_R |
| 89 | Thalamus_L |
| 90 | Thalamus_R |

##

## Table ST2 . Entropy of the output of each selected method (PHATE, PCA and Spectral clustering) applied on different levels of data processing, for 7 clusters

| **Method** | **Entropy (nats)** |
| --- | --- |
| PHATE on avalanche patterns | 5.62 |
| PHATE on avalanches | 5.90 |
| PHATE on source data | 6.19 |
| PHATE on normalised data | 6.20 |
| PCA on avalanche patterns | 5.89 |
| PCA on avalanches | 5.77 |
| PCA on source data | 5.99 |
| PCA on normalised data | 6.13 |
| Spectral clustering on avalanche patterns | 5.54 |

## Table ST3 . Entropy of the output of each selected method (PHATE and Spectral clustering for different numbers of clusters)

####

| **Method** | **Entropy (nats)** |
| --- | --- |
| PHATE (7 clusters) | 5.62 |
| Spectral clustering (3 clusters) | 5.22 |
| Spectral clustering (4 clusters) | 5.34 |
| Spectral clustering (5 clusters) | 5.44 |
| Spectral clustering (6 clusters) | 5.52 |
| Spectral clustering (7 clusters) | 5.54 |
| Spectral clustering (8 clusters) | 5.56 |
| Spectral clustering (9 clusters) | 5.76 |
| Spectral clustering (10 clusters) | 5.76 |
| Spectral clustering (11 clusters) | 5.76 |
| Spectral clustering (12 clusters) | 5.76 |
| Spectral clustering (13 clusters) | 5.76 |

## Table ST4 . Average of maximum correlations between PHATE and different algorithms (PHATE, Spectral clustering, PCA)

|  | **PHATE** | **Spectral clustering** | **PCA** |
| --- | --- | --- | --- |
| **PHATE** | 1.0 | 0.81 | 0.75 |
